# Non-sequential and multi-step splicing of the dystrophin transcript

**DOI:** 10.1080/15476286.2015.1125074

**Published:** 2015-12-15

**Authors:** Isabella Gazzoli, Irina Pulyakhina, Nisha E. Verwey, Yavuz Ariyurek, Jeroen F. J. Laros, Peter A. C. 't Hoen, Annemieke Aartsma-Rus

**Affiliations:** a Department of Human Genetics, Leiden University Medical Center, Leiden, the Netherlands; b Leiden Genome Technology Center, Leiden University Medical Center, Leiden, The Netherlands

**Keywords:** Capture-pre-mRNA-seq, Duchenne muscular dystrophy, exon blocks, intron removal, next generation sequencing, nested splicing, order of splicing, recursive splicing, splicing

## Abstract

The dystrophin protein encoding DMD gene is the longest human gene. The 2.2 Mb long human dystrophin transcript takes 16 hours to be transcribed and is co-transcriptionally spliced. It contains long introns (24 over 10kb long, 5 over 100kb long) and the heterogeneity in intron size makes it an ideal transcript to study different aspects of the human splicing process. Splicing is a complex process and much is unknown regarding the splicing of long introns in human genes.

Here, we used ultra-deep transcript sequencing to characterize splicing of the dystrophin transcripts in 3 different human skeletal muscle cell lines, and explored the order of intron removal and multi-step splicing. Coverage and read pair analyses showed that around 40% of the introns were not always removed sequentially. Additionally, for the first time, we report that non-consecutive intron removal resulted in 3 or more joined exons which are flanked by unspliced introns and we defined these joined exons as an exon block. Lastly, computational and experimental data revealed that, for the majority of dystrophin introns, multistep splicing events are used to splice out a single intron.

Overall, our data show for the first time in a human transcript, that multi-step intron removal is a general feature of mRNA splicing.

## Abbreviations

*DMD*: Duchenne Muscular Dystrophy gene
snRNPs: Small nuclear ribonucleoprotein particles
hnRNPs: heterogeneous ribonucleoproteins
SS: splice sites
BP: branch point sequence
ESE: exonic splicing enhancers
ISE: intronic splicing enhancers
ESS: exonic splicing silencers
ISS: intronic splicing silencers
RNA Pol II: RNA Polymerase II
RS: recursive splicing
5′RP: 5′ ratchetting point
3′RP: 3′ ratchetting point
ex-ex: exon-exon junction
FBS: Fetal Bovine Serum
P/S, penicillin/streptomycin
S: sequential
NS: non-sequential.

## Introduction

Splicing involves hundreds of proteins that coordinate the excision of introns from the pre-mRNA, joining the exons and resulting in mature mRNA transcripts. Multiple alternatively spliced transcripts can be produced from a single transcribed pre-mRNA molecule through a highly regulated process, and its disruption contributes to a large number of human genetic disorders that either directly cause disease or increase disease susceptibility.^1^ RNA splicing occurs after assembly of the spliceosome on the pre-mRNA, which includes splice site recognition and intron removal steps.^2^ Splice site recognition relies on the identification of exon/intron boundaries. This is achieved by 5 (U1, U2/U12, U4/U6 and U5) small nuclear ribonucleoprotein particles (snRNPs), together with more than 100 auxiliary proteins and *trans-*acting splicing factors (SR proteins and heterogeneous ribonucleoproteins (hnRNPs)).^3-5^ The correct recognition is supported by *cis*-acting splicing signals,^6^ such as the consensus donor (5′) and acceptor (3′) splice sites (SS), the branch point sequence (BP) and polypyrimidine tracts (PPT). Additional exonic or intronic splicing enhancers (ESE or ISE) and silencers (ESS or ISS) motifs can influence the inclusion or exclusion of an exon by recruiting *trans*-acting splicing factors. Intron removal is the result of 2 phosphoryl transfer reactions during the spliceosome assembly formation on the pre-mRNA, and the catalysis can only occur after the intron is transcribed. The precise excision of the intron results in the release of a lariat RNA^7,8^ and in 2 ligated exons.

It has recently been reported that the chromatin structure, the transcript elongation rate and the pausing of RNA Polymerase (Pol) II can contribute to the regulation of splicing.^9,10,11^ It has been established that splicing can occur co-transcriptionally, when the nascent transcript is still attached to the DNA through RNA Polymerase II,^12-15^ and/or post-transcriptionally, when splicing occurs after transcription has completed and the transcript has been transferred to a different nucleoplasmatic location, the speckles.^15^ Additionally, Vargas et al.^15^ showed that constitutive introns are mainly co-transcriptionally spliced, while alternative splicing may occur post-transcriptionally.^13,16-18^ The order of intron removal may confer an important regulatory layer for alternative splicing.

**Figure 1.**
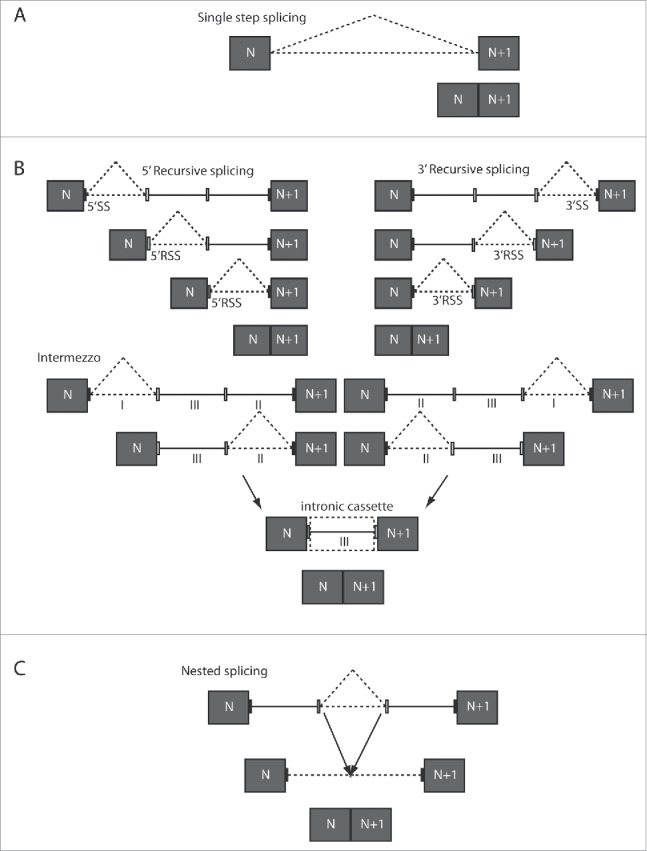
Single and multi-step splicing model. (A) Single step splicing. The intron is fully removed in one step using annotated 5′ and 3′ splice sites (black rectangles), to join neighboring exons (gray boxes, (N and N+1)). (B) Recursive splicing. In the case of 5′ recursive splicing (5′RS) or 3′recursive splicing (3′RS), the intron is spliced in multiple steps (ratchetting points), each starting from the 5′or 3′splice site (SS), respectively (left and right panel). Each step generates a new 5′ or 3′ recursive splice site (5′RSS or 3′RSS, white rectangles) respectively. A combination of 5′ and 3′ recursive splicing or intermezzo can also occur (lower panel). In this intermezzo splicing, parts I and II of the intron are first removed beginning from the 5′ or 3′ splice sites, leaving part of the intron (III) containing new unannotated 5′and 3′RSS (white rectangles), after which the final part of the intron is spliced. (C) Nested splicing. The first step(s) of intron splicing consist of removal of (an) internal part(s) of the intron using internal 5′ and 3′ splice sites (white rectangles). Subsequently, the remaining part of the intron is removed using the regular 5′ and 3′ splice sites that border the exon-intron boundaries (black rectangles).

For large introns, the precise excision and the efficiency of splicing may be hampered by the presence of multiple splice site-like sequences. Furthermore, the physical distance between donor and acceptor splice sites offers a challenge. It has been suggested that secondary RNA structure leads to juxtaposition of remote canonical donor and acceptor sites to facilitate identification and joining of splice sites,^19^ but additional mechanisms to facilitate splicing of long introns have been reported for invertebrates, such as intron removal in multiple steps (Fig. 1A-C).^20-23^

Recursive splicing can occur in different pathways (Fig. 1B). In the 5′ recursive splicing (RS) (Fig. 1B, left panel), a canonical donor splice site is spliced to an internal acceptor site, generating a 5′ ratchetting point (5′RP) from the juxtaposed exon and 5′ splice site sequences. A similar process can also take place at the 3′ splice site, but now an internal donor splice site is spliced to the canonical acceptor, to generate a 3′RP (Fig. 1B, right panel). This process can be repeated multiple times, creating 5′ or 3′ splice sites (SSs) that can be used as donor or acceptor splice sites in the next splicing step. Alternatively, 5′ and 3′ RP steps can generate an intermediate intronic cassette (intermezzo), which is removed in the last step of splicing (Fig. 1B, bottom panel). Finally, intrasplicing or nested splicing has been proposed as a third potential mechanism.^23,24^ Here the intron is first shortened by one or more internal splicing steps using internal donor and acceptor sites, and then in the final step what remains of the intron is spliced out using the canonical 5′and 3′ SSs upstream and downstream exons are joined (Fig. 1C).

Detailed studies on recursive splicing have been performed in Drosophila,^20-22,25^ but only few analyses, for single intron, have been done for human,^24,26^ and vertebrates.^19,27,28^

The dystrophin protein encoding *DMD* gene is the longest human gene (2.2 Mb). The coding regions represent only 0.7% of the gene, and the gene has exceptionally long introns (average 28 kb, size range 107 bp - 360 kb). In the1990s, evidence for co-transcriptional splicing for dystrophin transcripts was provided.^29^ This finding was expected, considering that full transcription of the gene takes an approximately 16 hours at an average elongation rate of 2.4 kb min^−1^. The size and complexity of the gene, containing 79 exons, long introns, 7 different promoters, 2 polyadenylation sites and numerous alternative transcripts, has always hampered characterization of the *DMD* transcriptome and detailed analysis of its processing. Indeed, only recent experimental evidence of an internal lariat of dystrophin intron 7 suggested that this long intron (110 kb) might undergo to nested splicing.^24^

In the last few years, the development of next generation sequencing technologies has opened a new horizon for the detailed analysis of transcript processing. The *DMD* gene is an excellent candidate for in depth analysis of the relationship between intron length and the order of intron splicing, as well as the occurrence of splicing of the large introns in multiple steps.

Here we present detailed analysis of dystrophin pre-mRNA intron splicing using targeted paired end sequencing of transcripts, Capture-pre-mRNA-sequencing. We provide evidence that the order at which introns are removed is not consecutive, leading to the formation of blocks of exons flanked by unspliced introns. We further show the occurrence of multi-step splicing in many dystrophin introns, and show for the first time the characterization and validation of recursive splicing in the dystrophin transcript.

## Results

To investigate the splicing of the dystrophin transcript in detail, we performed Illumina HiSeq paired end sequencing on pre-mRNA isolated from 3 differentiated human muscle cell lines. To enrich for pre-mRNA, we isolated RNA from cell nuclei. Since dystrophin is expressed at very low levels, we enriched for dystrophin pre-mRNA using a customized library that consisted of biotinylated probes covering all exons, introns, annotated promoters and UTRs of *DMD* as well as 3 control genes, excluding repeat masked areas. The captured cDNA was sequenced using Illumina HiSeq 2000 to generate paired-ends reads of 100 nt each producing between 8.5 and 11.5 million of reads for the different samples (Table S1).

Whereas many next generation sequencing analysis pipelines are available for analyzing mRNA-seq data, a method for analyzing pre-mRNA has not been reported. To facilitate the analysis of our dataset, we developed a novel pipeline (SplicePie).^30^

### Sample preparation

A library was generated from genomic DNA of one of the cell lines and sequenced, confirming coverage in all introns and exons, probes specificity and efficient capture sequencing across all regions in the *DMD* and control genes. Analysis of the DNA sample (CaptureSeq 6) revealed a 900-fold enrichment with 56% of reads mapped to *DMD* out of 11.5 million of uniquely mapped reads (Table S1). As expected for DNA, equal coverage of exons and introns was observed with the exception of repeat areas in which no probes were designed.

For the pre-mRNA splicing analysis, PCR analysis confirmed the absence of DNA from the RNA that was isolated from the intact nuclei of differentiated myotubes. Comparison of the fragmented and sonicated cDNA libraries from nuclei of a differentiated healthy muscle cell line (7304) revealed that the number of reads mapping to the *DMD* gene was 240,601 (1.1%) and 2,245,758 (12.7%) for the fragmented and the sonicated samples, respectively. Furthermore, for the fragmented sample, we did not observe a uniform coverage profile of the exonic and intronic regions (Fig. S1), while the coverage was much more uniform for the sonicated sample. For the following experiments random primers and sonication post-cDNA synthesis was used as sample preparation.

When analyzing the pre-mRNA, we observed clearly higher coverage in exonic regions compared to intronic regions. This could be due to the presence mixtures of pre-mRNA and co- or posttranscriptionally spliced mRNA in the nucleus. We therefore classified our paired-end reads into 3 categories: reads originating from post-, intermediate- or pre- splicing phases. The post-splicing category contained paired end reads spanning 2 different exons (ex∼ex), one exon and one exon-exon junction (ex∼(ex-ex)) or 2 exon-exon junctions (ex-ex)∼(ex-ex), implying completed splicing events. The intermediate-splicing category included read pairs where one read spanned an exon-intron boundary (ex-in; in-ex) or maps to an intron, while the other spans an exon-exon junction (ex-ex). Additionally, paired end reads mapping to the same intron or different introns (in∼in), but with a mapping distance (between the 2 reads) exceeding the library insert distance belong to the intermediate group. This category contained reads reflecting the initial and ongoing splicing events within one or multiple introns. The pre-splicing category contained paired end reads where one or both ends were mapped to the intronic sequences or exon-intron junctions (i.e. both reads did not cover exon-exon junctions), thus reflecting unspliced fragments. For the DNA sample, 99.9% of reads mapped to the pre-splicing group, which was expected because the DNA sample of course does not contain exon-exon junction reads. For the 5 pre-mRNA CaptureSeq an average of 81% of mapped reads belonged to the pre-splicing group, suggesting pre-mRNA enrichment. Only 1.5–3.8% of read pairs fell in the intermediate category, probably due to the fact that splicing is a relatively fast process. The distribution of the reads over the different categories was similar for all samples.

### Reproducibility of the method

We generated libraries from captured pre-mRNA isolated from muscle cell line 7304 after 8 days (CaptureSeq 1 and 2, biological replicates) or 14 days of differentiation (CaptureSeq 3), and from muscle cell lines KM155 (CaptureSeq 4) and 8220 (CaptureSeq 5) after 8 days differentiation using the optimized protocol. Between 749,012 to 6,140,259 reads mapped to the human *DMD* reference sequence (6.9–65.8% of the total number of reads obtained) (Table S1). To allow comparison between different samples, the coverage was normalized by the number of reads mapping to *DMD* exons.

When analyzing the coverage of introns, we removed regions containing annotated promoters, UTRs, and expressed RNAs from the analysis (Table S2), because they can have high coverage unrelated to the splicing process. We did not normalize for the GC content, because we did not observe a correlation between the intron coverage and GC percentage of introns or the GC percentage of the probes. To assess the presence of the 5′-3′ bias in the *DMD* coverage data (the bias which is reflected in coverage decreasing toward the 3′ end due to technicalities such as incomplete sequencing transcripts, but not biological reasons), we explored the behavior of intronic coverage over the gene across 3 cell lines. We performed linear regression on the median coverage values of the 78 introns of pre-mRNA samples. We discovered that none of the linear regressions appeared to be significant (Km155, p-value = 0.64; 8220, p-value = 0.08; 7304, p-value = 0.39), indicating the absence of a 5′-3′ bias. This is a first indication for co-transcriptional splicing. When splicing would occur only after completion of transcription, the presence of nascent transcripts would lead to a higher representation of introns at the 5′ end of the transcript. High intronic coverage was observed for CaptureSeq 4 and 5 that were derived from 2 different cell lines (Table S1). For CaptureSeq 1 and 2 (biological replicates of the third cell line) we observed that the percentage of reads mapping to the *DMD* gene was lower, while CaptureSeq 3 (same cell line but differentiated for 14 instead of 8 days) had a higher coverage of *DMD* introns. The biological variation observed between different cell lines and even between different biological replicates of the same cell line is to be expected. Dystrophin expression is initiated on differentiation and depends on the amount of myogenic cells in a culture and the confluence of the cells when differentiation is initiated. Sample preparation could be another cause for variation. However, this did not influence the coverage in this experiment, since no large deviation was observed between the percentage of duplicate reads for *DMD* (Table S1) or the control genes between biological replicates or between different cell lines.^30^ Out of the reads mapping to the dystrophin transcript for the 5 capture-pre-mRNA-seq, 13–30% covered exonic sequences while 67–82% covered intronic sequences.

**Figure 2.**
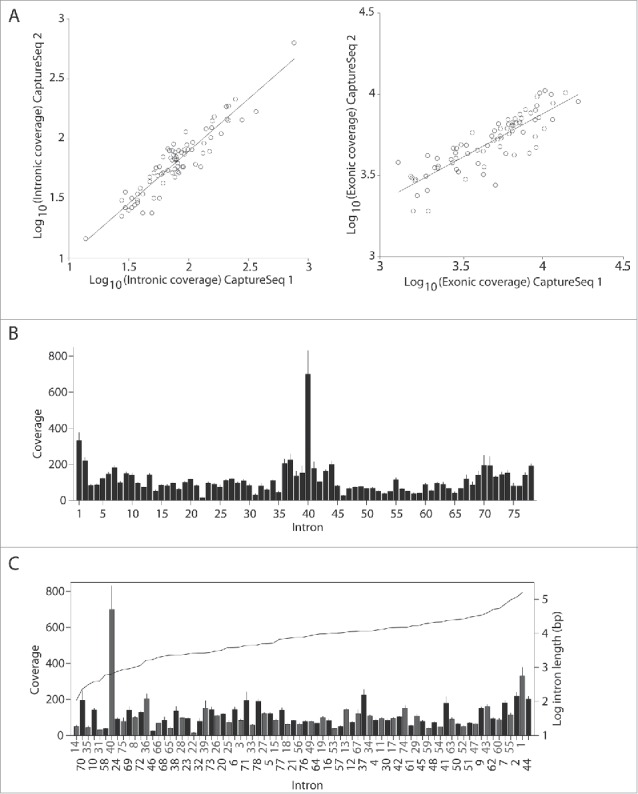
Graphical representation of the intronic coverage. (A) Scatter plots and regression lines showing a high correlation of the median normalized intron coverage (left panel, R = 0.94, P = 5.1. 10^−36^) and exon coverage (right panel, R= 0.85, P= 4.3. 10^−23^) for 2 biological replicates (CaptureSeq 1 and 2) from the same cell line 7304. Logarithmic scales are used for both axes. (B) Bar graph showing the average normalized coverage of each intron for the 3 cell lines (error bars reflect the standard error of the mean). (C) The same bar graph as shown in 2b, but now introns are sorted by length and represented in gray or black (ascending, length represented by the black dotted line and right y-axis (^10^log-scale).

To assess the reproducibility of *DMD* capture cDNA seq analysis, we compared the results from 2 biological replicates, performing 2 independent experiments with cell line 7304. This (Fig. 2A left panel) showed a high correlation for intronic coverage (R= 0.94, P= 5.1. 10^−36^), indicating that the experimental procedure is consistent and reproducible, which was further confirmed by a significant correlation in exonic read distributions (Fig. 2A right panel, R = 0.85, P = 4.3. 10^−23^).

Two additional cell lines (KM155 and 8220) were analyzed to confirm the reproducibility of the method and to confirm the results in different sources of material. Correlation of the intronic coverage was observed between the 3 cell lines (Fig. S2A-B-C). Furthermore, the profiles along the whole gene showed the same distribution pattern and similar depth (Fig. 2B). Based on these findings no additional biological replicates were considered.

### Sequential and non-sequential splicing

**Figure 3.**
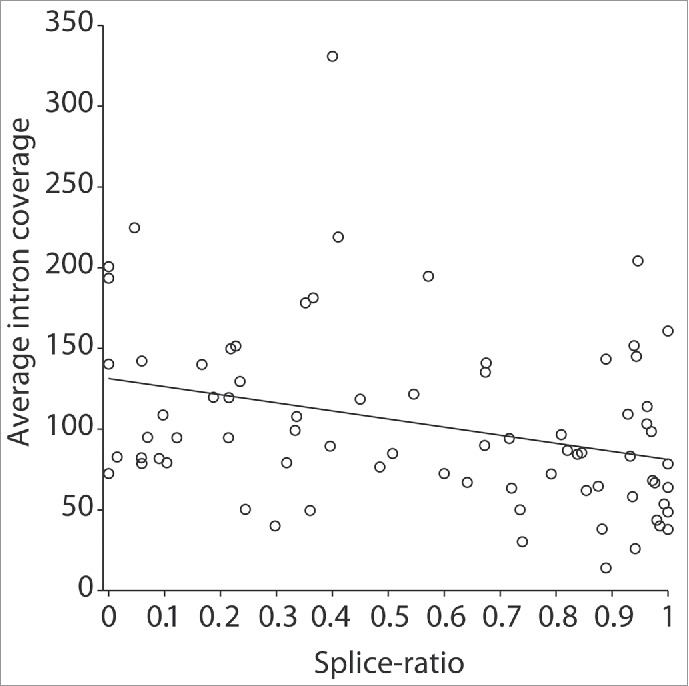
Scatter plot of the average intron coverage (y-axis) ***vs***. the splice-ratio (x-axis) of each intron. An inverse correlation between the 2 methods is observed (r =− 0.32, p-value = 0.0043): lower coverage (relatively fast splicing) is associated with a higher splice-ratio, indicative of sequential splicing.

We reasoned that the intronic coverage would correlate with relative speed of intron removal, *i.e.*, introns that are spliced out quickly are expected to show low coverage, while introns that are spliced out slowly are expected to show higher coverage. Since there is a large variation in the length of introns in dystrophin transcript, we first addressed whether the coverage was proportional to the intron length. We defined intronic length as the amount of nucleotides covered by the probes and then subtracted sequences containing annotated promoters, UTRs, micro-RNAs for each intron and assessed the read density of the remaining intronic sequences. No significant correlation between intron length and coverage (Fig. 2C) indicating that short introns are not spliced before long introns. Rather, these results suggested that the introns are non-sequentially spliced. Therefore, some introns may be removed only after downstream introns have been removed and the splicing does not follow a strict 5′-3′ order. Nevertheless, since transcription of the complete dystrophin transcript takes ∼16 hours, it is likely that a very slowly spliced upstream intron is spliced out before a very quickly spliced intron further downstream, simply because the downstream intron is produced hours later than the upstream intron. Therefore, we analyzed the relative order of intron removal in groups of 5 introns, using a sliding window of 3. For every group of 5 introns, each intron was classified as fast, intermediate or slow. A low depth of coverage may represent quickly spliced introns (normalized coverage <90), while a higher depth (normalized coverage >130) may reflect slow splicing. A small group of introns with coverage between 90 and 130 were defined as ‘intermediate’. The classification of introns was very similar for each of the 3 cell lines showing a strong indications that several downstream introns were removed before upstream introns, and as a consequence of this, blocks of exons that were flanked by slowly spliced introns were identified. Considering that intronic depth could also account for reads spanning putative lariats and non-annotated pseudoexons, we used a second analysis method to confirm out findings, using paired end analysis, where one read spans an exon-exon junction and the second read falls in an intron, thus excluding reads spanning lariat forms. Sequential and non-sequential splicing events were thus corroborated by the analysis of paired-end reads from the intermediate-splicing category. To determine the nature of splicing of each intron, we considered intron (n) as a starting point. If intron (n) is spliced sequentially (S), it would be spliced before intron (n+1), leading to read pairs where one end would cover the ex-ex junction (ex_(n)_-ex_(n+1)_) and the other read would align to the flanking downstream intron (n+1) (Fig. S3A). Alternatively, a non-sequential (NS) splicing would result in the splicing of intron (n+1) before intron (n). This would be reflected by paired-end reads in intron (n) and in the exon-exon junction of the 2 exons immediately downstream of intron (n), (ex_(n+1)_-ex_(n+2)_) implying the presence of an unspliced intron. We defined the splice-ratio for any given intron as the number of reads suggestive of sequential splicing, divided by the sum of the reads suggestive of sequential splicing and those reads suggestive of non-sequential splicing. Intron were classified as being sequentially spliced when the splice-ratio was between 0.5 and 1, while introns with a splice-ratio below 0.5 were classified as non-sequential. For five introns out of 78, splice-ratios were slightly above or below 0.5, and classified as intermediate. We discovered high pair wise correlation between the splice ratios in pre-mRNA samples between the 3 cell lines, after calculating Pearson correlation coefficient: 0.82 between cell lines KM155 and 8220; 0.85 between cell lines KM155 and 7304; 0.82 between cell lines 8220 and 7304. We also observed a correlation between the intron coverage and the splice-ratio values (Fig. 3), where introns classified as non-sequential based on the splice-ratio showed higher coverage (indicative of slower splicing) than introns classified as sequential (r=−0.32, p-value=0.0043). The fact that the intron coverage analysis may also have included excised lariats, while the paired-end analysis does not, may have prevented the correlation from being better than it is now.

**Figure 4.**
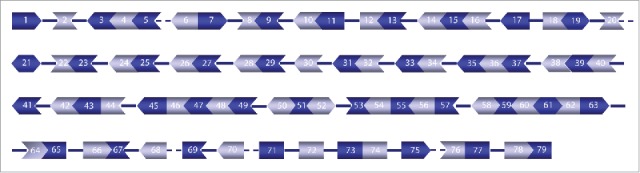
Representation of sequential and non-sequential splicing of the dystrophin transcript. Solid and dotted lines between 2 exons indicate preferential non-sequential splicing (slow and intermediate, respectively), while sequentially spliced introns are not shown. The exon shape reflects the phasing of exons based on the partition of the codon triplet at the beginning of the exon (< indicates the exon ends at the end of a triplet, while for | and > the triples are split). Two colors have been used to improve visualization.

Fig. 4 shows a graphical depiction of sequential and non-sequential splicing, (and intermediate stage of few introns), of the dystrophin transcript. We propose the presence of blocks of exons, where 3 or more exons are joined flanked by slowly removed introns.

**Figure 5.**
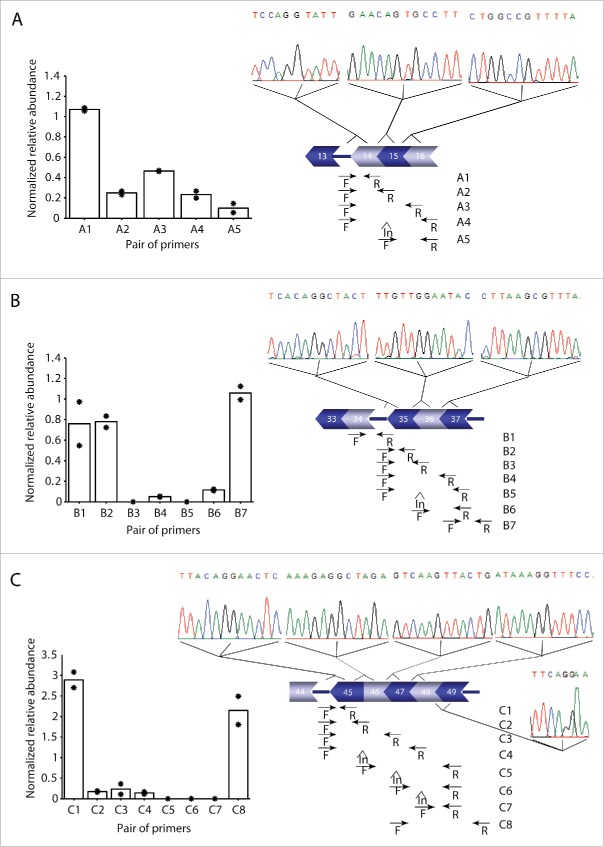
Experimental validation of 3 of the predicted exon blocks. (A) Intron13-Exons14-15-16. (B) Exons 33-34-Intron 34-Exons 35-36-37. **(C)** Intron 44-Exons 45-46-47-48-49-Intron49. The same analysis has been performed for the 3 predicted cases. For each case the left panel (bar plot) shows (qRT-PCR) results representing relative abundance ( to the first primer pair) of the spliced and unspliced introns using primer pairs in an intron and downstream exons, or exon-exon junctions (locations shown in the panel on the right). The following primer pairs were used in the qRT-PCR: A1(In13F-Ex14R), A2(In13F-Ex14/15R), A3(In13F-Ex15/16R), A4(In13F-Ex16R2), A5(In14F-Ex16R2), B1(Ex33/34F-In34R), B2(In34F3-Ex35R), B3(In34F3-Ex35/36R), B4(In34F3-Ex36/37R2), B5(In35F-Ex37R), B6(Ex35/36F-In37R), B7(Ex36/37F-In37R), C1(In44F-Ex45R), C2(In44F-Ex 45/46R), C3(In44F-Ex46/47R), C4(In44F-Ex47/48R), C5(In45F-Ex48/49R), C6(In46F-Ex48/49R), C7(In47F-Ex48/49R), C8 (Ex46/47F-In49R). *HPRT* expression was used for normalization. The qRT-PCR values are based on the average levels of 2 independent cell lines (individual levels (based on triplicates) are indicated with asterisks). The amplicons A1, B2, C1 were used as internal PCR efficiency controls. The detection of one amplicon (B3) was hampered by very low efficiency of the primer, mainly due to the hairpin and dimer structures. Attempts with an alternative primer did not improve the PCR efficiency. Sequential splicing of introns 14 and 15 is supported by amplicons generated with the pair of primers A2 and A3. Additionally, amplification with the forward primer in intron 14 and the reverse primer in intron 16 (A5), showed partial splicing of intron 14, supported by the difference in the relative abundance between A2 and A3. Sanger sequencing was used to confirm the 3 predicted exon blocks and the electropherograms (right panel) show the junction sequences for each case (intron-exon, exon-exon or exon-intron boundaries/junctions detected in a single fragment). The schematic illustration on the low side of the electropherogram shows the predicted exon block and the location of the primers used for qRT-PCR and Sanger sequencing PCR.

We experimentally validated the presence of these blocks using qPCR and Sanger sequencing analysis to confirm the presence of dystrophin pre-mRNA transcripts containing an upstream intron, when downstream exons had already been joined (Fig. 5). With primers pairs (Table S3) that were designed to cover unspliced introns, exon-exon junctions and using predicted quickly spliced introns as a negative control, we confirmed exon block 14-15-16 (Fig. 5A). Using qPCR, we confirmed that intron 15 was spliced before intron 13. Additional evidence of the non-sequential removal of intron 13 was obtained using a forward primer in intron 13 and reverse primers on the exon 14–15 boundary. The relative abundance of the product with the exon 14–15 primer was lower than that obtained with the exon 15–16 primer, suggesting that intron 15 is spliced earlier than intron 14. This finding was supported by the presence of an additional PCR fragment that included intron 14 using primers in intron 13 and exon 16. Conventional PCR and Sanger sequencing using a combination of primers in intron 13 and exon 16 showed the junctions of the 3 exons (14-15-16), confirming the predicted non-sequential splicing of intron 13.

Likewise, we observed a block of exons 33–34 and exons 35-36-37 (Fig. 5B). In this case our data was supportive of non-sequential splicing of intron 34, as we did not detect introns 33, 35 and 36, while intron 34 was still present, albeit with low abundance. Quick removal of intron 35 was validated as well, since we were unable to generate a PCR fragment using a primer pair in intron 35 and exon 37. Sanger sequencing confirmed the presence of a transcript containing introns 34 and 37, but without introns 33, 35 and 36.

Finally, a similar approach was used to test the third exon block including exons 45-46-47-48-49. As shown in Fig. 5C, we were able to detect fragments using the forward primer in the intron 44 and reverse primers in exons 45–46, exons 46–47 and exons 47–48. Using forward primers in introns 45, 46 and 47 combined with reverse primers in exon-exon junction 48–49, no signal was detected, confirming these introns are indeed removed quickly. Sequential splicing of introns 47 and 48 was also shown using primers spanning the junction between exons 46–47 and intron 49. Furthermore, Sanger sequencing confirmed the exon block from exon 45 to 49, between the unspliced introns 44 and 49, validating that intron 45, 46, 47 and 48 can be spliced before intron 44.

In addition, we validated few more predicted sequential and non-sequential events directly by qPCR and Sanger sequencing (Fig. S3B), showing splicing of intron 8 before intron 7, as well introns 50 and 51 spliced before intron 49.

### Recursive and nested splicing

Since *DMD* introns are remarkably long, we hypothesized that multi-step intron removal, such as recursive and nested splicing previously identified in *Drosophila*, could occur during the splicing of *DMD* transcripts (Figs. 1B, C).

To identify potential recursive and nested splicing events in an unbiased way, we analyzed split reads and first filtered out split reads that aligned to exon-exon junctions or mapping within 50 nucleotides to an exon junction to maintain only split reads representing a splicing event with a non-annotated splice donor and/or acceptor site. We generated a matrix that included coordinates of the 2 genomic positions for each pair of donor and acceptor sites and the detected number of split reads supporting the combination. As intermediate splicing events are difficult to detect and may be rare, we jointly analyzed all split reads from the 7304 cell line. We only included events present in all 3 different cell lines to avoid observations that were a consequence of PCR duplicates and to provide stronger support for the genuine presence of these intermediate splicing events. Using this filter, we identified 414 splicing events (Table S4), 35% of which could be classified as potential recursive splicing, including 5′RP (18%), 3′RP (17%). Splicing events were observed at beginning, in the middle or at the end of the intron, and were independently of intron length. We also found 266 events (65%) indicative of nested splicing. Notably, for 27 introns we identified more than one type of events. This could indicate complementary or independent splicing mechanisms affecting the same or different transcripts, respectively, speculated to be due to RNA secondary structures. Finally, for 31 introns we established single step splicing (Fig. 1A and Table S5).

**Figure 6.**
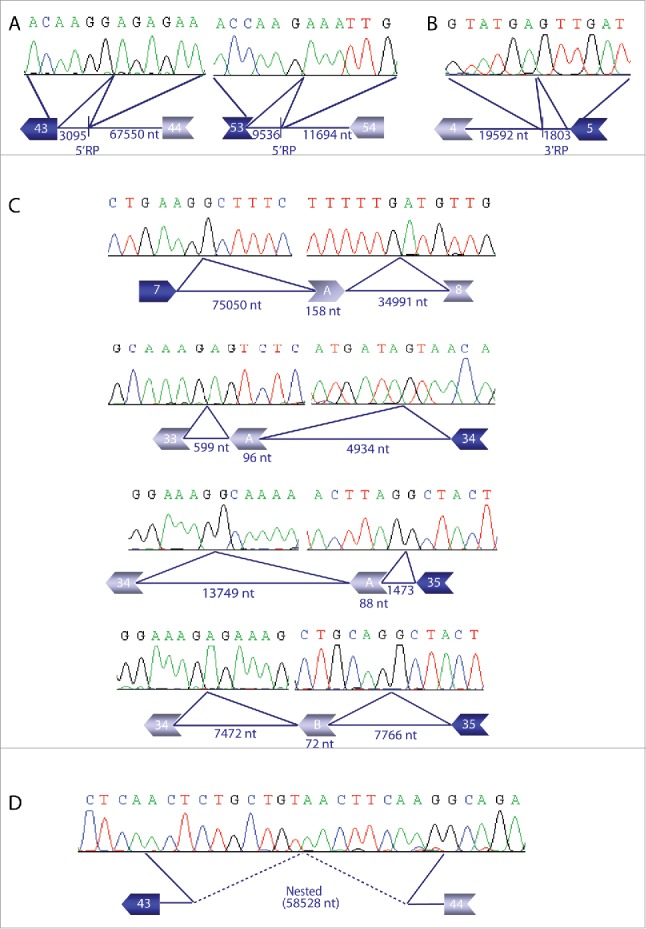
Examples of the experimental validation results of different types of intermediate splicing. (A) Partial splicing of 3095 and 9536 nucleotides (nt) in introns 43 and 53, respectively, using a 5′RP, are reported in the upper panel. For introns 4, partial splicing of intron of 1803 nt used 3′RP (B). Each electropherogram shows the last 6 nucleotides of the exon joined to internal intron sequence as consequence of partial intron removal. (C) Four preselected intermezzo events have been validated. In intron 7, a sequence of 158 nt (intermezzo A) was joined to the flanking exons 7 and 8, whereas another intermezzo event involving 96 nt was detected in intron 33. An area of 88 bp for intermezzo A and 72 bp for intermezzo B were identified between exons 34 and 35. (D) In intron 43, we predicted nested splicing resulting from partial splicing (>58kb) of the intron. Two predicted genomic positions are indicated by blue continuous lines. Retention of 9 nucleotides in each side of the split read (identified during the validation experiment) is reported between the continuous and the dashed lines. Joined point of the spliced intron is represented by a dashed line.

We tested 13 predicted events and performed RT-PCR amplifications across the split reads to detect the breakpoints using pre-mRNA from 2 libraries, followed by Sanger sequencing. We could validate 8 out of 13 events as shown in Fig. 6. This level of success was higher than anticipated, given that Capture-pre-mRNA-seq is a much more sensitive method compared to the standard RT-PCR. We chose 5′RP events identified in introns 42, 43 and 53 for the experimental validation. In introns 43 and 53, we confirmed the predicted 5′RP events, generating a spliced sequence of 3095 and 9536 bp, respectively. In both cases, sequencing of the expected PCR products (Fig. 6A) showed the junction of the exon 43 or 53 and the 5′ ratchetting point.

A similar approach was used for the validation of 3′RP in intron 4, 25, 45, 53, 45. A predicted 3′ recursive points in intron 4 was confirmed by Sanger sequencing (Fig. 6B). For some selected events, it was not possible to detect the breakpoint sequence. Furthermore, a few of the selected 5′ and 3′RP sites were revealed to be intermezzo recursive splicing events, where the 5′ and 3′ RP sites were used as donor and acceptor splice sites. Intermezzo splicing occurs when upstream and downstream parts of an intron are spliced, leaving the internal area joined to the flanking exons. Theoretically, such an intermezzo intron could also be an alternative exon. Therefore, we amplified cDNA from pre-mRNA and cytoplasmic mRNA, arguing that intermezzo introns should not be present in cytoplasmic RNA. We could validate the intermezzo event for introns 7, 33 and 43 (present in nuclear RNA but not in cytoplasmic RNA) by PCR and Sanger sequencing (Fig. 6C). Interestingly, for intron 43 we detected 2 intermezzo events. No split reads spanning both intermezzo events were found, suggesting that only one intermezzo is used at a time. For the selected nested splicing event in intron 43, we predicted 2 genomic positions based on split reads. Sanger sequencing of the PCR product (Fig. 6D) showed the removal of 58,528 nucleotides from the intron. We observed the retention of 9 nucleotides on each side of the predicted breakpoint. However, this retention could be due to misalignment of partial repeat sequences (TCAA) on both sides and the fact that we could pinpoint the removal of 58 kb by RT-PCR confirms this nested splicing event.

### Motif analysis

**Figure 7.**
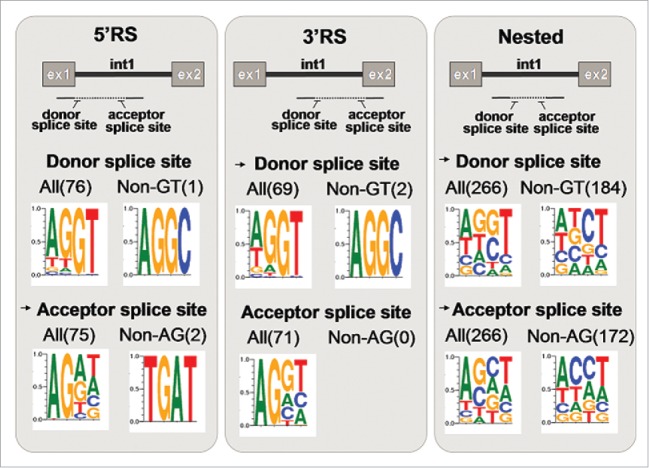
Motif analysis: sequence logo of the donor and acceptor splice sites involved in the recursive and nested splicing. On the first and second left panels, 5′and 3 recursive splicing (RS) are represented by the split read spanning the exon (1 or 2, respectively) and the middle part of intron 1. On the right panel ( nested splicing) the split read spans part of the internal sequence of intron 1. The beginning and the end of the dotted line show the positions of the donor and acceptor splice sites involved in the splicing step of intron 1. Non-annotated donor and acceptor splice sites are indicated with a black arrow. Four nucleotides, including 2 from each splice site and 2 upstream of the donor and 2 downstream of the acceptor have been used to define the sequence logo. The 5′ and 3′RS display a clear preference for the consensus splice site motifs. For nested splicing, sequences of both non-annotated splice sites display no particular consensus motif.

We evaluated the motif of the areas involved in recursive and nested splicing by analyzing sequence conservation of the newly detected donor and acceptor splice sites and the 2 nucleotides upstream and downstream of these sites, respectively. As shown in the Fig. 7, for the 5′and 3′ recursive splicing (RS) we observed AG and GT as the most frequent motifs for the intra-intronic (non-annotated) acceptor and donor, respectively, showing most 5′ RS and 3′RS use canonical splice site motifs (97% of acceptor sites and 95 % of donor sites). In case of the nested splicing events, no clear consensus motif could be distilled for the non-annotated donor and acceptor splice sites.

## Discussion

The use of target enrichment in combination with deep sequencing of cDNA offers an opportunity to study rare splicing events.^31^ However, the identification of this small portion of intermediately spliced transcripts relies on the accuracy and sensitivity of the analysis and source material. Although RNA-seq is an appealing approach to study dystrophin transcripts, the use of total mRNA is not suitable, as the vast majority of sequence reads would reflect spliced transcripts. While this would be useful to identify e.g. alternative splicing or poly-adenylation events, it would preclude the analysis of intron removal and transcript processing, because dystrophin is expressed at low levels, and the pre-mRNA transcripts would be in very low abundance. As such, it is unlikely that these transcripts would be picked up during mRNA-seq analysis. Here, we present a similar approach of deep sequencing of a specific target gene using pre-mRNA isolated from nuclei as input material (Capture-pre-mRNA-seq). This method provided us an unprecedented way of understanding the details and mechanism of the splicing of *DMD* gene. Using subcellular RNA fractions and a solution hybridization library has been engaged before in RNA-seq analysis for human genes,^13,32,33^ but for the first time the combination of these 2 methods is applied to a single gene with the aim of dissecting the splicing of large introns. Additionally we have previously developed a computational pipeline, SplicePie, to comprehensively analyze and detect intermediate splicing products.^30^

Considering the complexity of the *DMD* gene, with co-transcriptional activity varying intron sizes (between 107 bp-360,000 bp), it was hypothesized that the order of intron removal was not sequential. Based on our findings using 2 independent data analysis methods using SplicePie and experimental validation we confirmed that the order of intron removal does not follow a consecutive 5′-3′ direction. Moreover, the relative speed of intron removal is not dependent on intron length, as initially hypothesized. Others have reported that the intron length does not influence the order of intron excision.^9,13^ Additionally, studies in other genes have shown that downstream introns can be spliced before upstream introns.^34-36^ The speed and efficiency of intron removal may be regulated by co-transcriptional activity.^37-39^ Since it is now apparent that intron removal does not always follow “first come, first served” model^40^ a “first served, first committed” model has been proposed that takes the speed of the RNA Pol II activity into account,^18^ where the rate of RNA Pol II elongation affects the speed of splicing factor recruitment to different splice sites, facilitating introns excision independent of the co-transcriptional direction and strength of the splice sites. The identification of non-sequential intron removal in *DMD* has been supported by the evidence that exons can be joined to generate what we defined as “exon blocks”. These joined blocks of exons flanked by introns are intermediate steps of the final mature RNA. Notably, our findings reflect a propensity of sequentially or non-sequentially spliced introns.

Mutations in the *DMD* gene underlie a severe muscular dystrophy, Duchenne muscular dystrophy and a milder muscular dystrophy, Becker muscular dystrophy, depending on whether mutations disrupt or maintain the open reading frame, respectively.^41^ Antisense oligonucleotide-mediated exon skipping is a therapeutic approach that aims to restore the reading frame for *DMD* transcripts for Duchenne patients to convert a severe phenotype into a milder one.^42^ Our findings explain previous findings, where the use of one or 2 antisense oligonucleotides (AONs) could result in the skipping of multiple exons. Indeed as previously reported,^43,44^ all tested AONs targeting exon 8 resulted in skipping of both exon 8 and 9, and here we show experimental validation for this. Another notable example is the exon 45–51 skipping.^45^ The exon 45–55 area is a hotspot for DMD deletions,^46-48^ and skipping these 11 exons would be beneficial for 40% of patients.^49^ So far inducing exon 45–55 skipping has been challenging for human *DMD*,^45^ but successful in the murine *Dmd* gene.^50^ Nevertheless, this required a mix of 10 different antisense oligonucleotides, which is untenable for clinical development based on translational and regulatory challenges. Our data on exon blocks however, provides insight in how to induce exon 45–55 skipping with less antisense oligonucleotides, by targeting the blocks rather than individual exons. Based on our data exon 45–55 skipping could be technically challenging since intron 44 is spliced non-sequentially, while intron 55 is not. However, by targeting the exon blocks involving exon 45–49, exon 50–52 and exon 53–57 it might possible to achieve in-frame exon 45–57 skipping.

Additionally for the first time, we found evidence for recursive and nested splicing for different *DMD* introns, employing different ways of multi-step splicing and more likely in the long introns (Fig. 1). Previous evidence from another long human gene, *UTRN*, indicated that intron length did not correlate with the time required of splicing.^9^ Additionally the authors showed that introns in the range of 1.2kb to 240kb were spliced within 5–10 minutes, suggesting that the physical distance between donor and acceptor splice site is kept small by mechanisms like recursive splicing or alternatively, by the associated 5′splice site to the C-terminal domain of the RNA polymerase II, increasing the efficiency of splicing and reducing the time. Currently, Sibley et al.^27^ reported recursive splice sites with high incidence in long introns in all vertebrates and most of the 435 identified in the longest human genes.

Our data showed that the average size of ‘single step spliced introns’ is 6.4 kb (107– 38,368 bp), while introns spliced via multi-step splicing introns are on average 40 kb long (650 – 248,401 bp), suggesting that, as anticipated, multi-step splicing involves generally longer introns. In fact, this difference was significant (Wilcoxon signed-rank test *P* 2.6 10^−8^). Likewise, we observed that single step introns were primarily spliced sequentially (19/31), while multi-step introns were primarily spliced non-sequentially (24/47), however, this enrichment is not significant (Fisher's exact test P 0.95). Finally we tested whether multistep splicing or non-sequentially spliced introns occurred more frequently at the beginning, middle or end of the gene, but this was not the case (Fisher's exact test, P 0.9). Notably, 72% of introns in the first half of the gene were spliced in multiple steps and non-sequentially, while in the central region (exon 45–55) introns were generally spliced in multiple steps but in a sequential manner, while 65% of introns in the last part of the gene were spliced sequentially in a single step.

We assumed the 31 introns, for which no evidence of multi-step splicing was observed, were spliced in a single step, but since the frequency of reported recursive and nested events was sometimes low, we cannot exclude the possibility that some of these introns are removed in multiple steps. For the remaining 47 introns, evidence for multi-step splicing was found for each of the 3 tested human skeletal muscle cell lines, including 5′ and 3′ recursive splicing and nested splicing, which could be validated by RT-PCR analysis. Additionally, during the experimental analysis a few of the predicted 5′ and 3′RS turned out to be “non-annotated” donor and acceptor splice sites of intermezzo events. This suggested that some of the other predicted recursive events were also intermezzo splicing events.

Recently, 2 independent groups reported evidence of recursive splicing in few different human genes.^27,28^ Both works provided experimental validation of intermezzo splicing, where the inclusion or exclusion of a “recursive exon” could be detected in the mRNA or was part the last step of splicing. A previous case of nested splicing was also reported in *DMD* intron 7.^24^ However, this event was not detected in our data set, not even when taking only single cell lines into account. This discrepancy can be due to a different method of identification. Suzuki used PCR primer pairs with downstream forward primers and upstream reversed primers to generate fragments from lariats removed during nested splicing in RNA isolated from a single cell line. By contrast, we analyzed multiple cell lines with capture-pre-mRNA-seq. It is possible that the events reported by Suzuki occurred in our cell lines, but we were unable to pick them up, or alternatively that they occurred only in the cell line he used.

Although only the results of the *DMD* gene have been reported here, extensive analysis has shown non-sequential and recursive splicing in one of our control genes (*FXR1*) in 5 capture libraries, which could be validated experimentally.^30^ This suggests that recursive splicing may constitute a common mechanism to remove larger introns or introns from transcripts undergoing complex splicing pattern.

Considering the rarity of these events, good reproducibility of most of the findings have been achieved in all 3 different cell lines, providing efficiently insight in the splicing, without requiring additional replicates along with an increased sequence depth.

Motif analysis of sequences involved in multi-step splicing events for *DMD* revealed that recursive splicing relies primarily on known 5′ and 3′ consensus splicing motifs. By contrast, no real motif could be identified for nested splicing events. For 63 events we identified conventional GT-AG sequences, this was not the case for the majority of events. We studied the frequency of canonical and common non-canonical splice sites as listed by Burset et al.,^51^ for our nested splicing events. The canonical GT-AG (donor-acceptor) was detected for 23.6% of nested splicing events (Fig. 7). Other listed events found were GT-TG (1.5%), GC-AG (3%), GA-AG (0.4%), GT-AC (0.8%) and AT-AG (1.1%), while GT-CG and AT-AC motifs were not found. Notably, CT-AC (not listed by Burset) was detected for 17.6% of our nested splicing events. However, for the majority of events (>50%) no clear motif could be detected, suggesting that a different, as yet unknown mechanism is employed for nested splicing.

In conclusion, our work provides splicing analysis of the dystrophin transcript at an unprecedented depth, shows evidence for non-sequential removal of introns, generating exons block, and multi-step intron removal as a common mechanism for dystrophin intron splicing.

## Materials & methods

### Cell culture

All experiments were conducted using 3 immortalized control muscle cell lines (7304, Km155, 8220) generated by Zhu et al.^52^ that were propagated and differentiated as described previously.^53^ In short, cells were cultured in Skeletal Muscle cell medium ((PromoCell GmbH, Germany) with 20% Fetal Bovine Serum (FBS), 1% of penicillin/streptomycin (P/S; Gibco-BRL) at 37°C in a humidified atmosphere with 5% CO_2_. One hundred million cells were seeded and, as they approached a confluence of 70%, proliferation medium was replaced with differentiation medium (DMEM, 2% horse serum, 1% P/S) to obtain multinucleated myotubes. Cells were allowed to differentiate for 8 or 14 days.

### Subcellular fractionation and RNA extractions

Nuclear and cytoplasmic fractions were separated as previously described,^54^ with minor changes. All steps were carried out on ice.

At eight and 14 days after initiating differentiation, cells were harvested via trypsinization and centrifuged for 10 min at 2000*g*. After washing twice with cold PBS, the pellet was resuspended in 2 ml ice-cold sucrose buffer I (0.32 M sucrose, 3 mM CaCl_2_, 2 mM magnesium acetate, 0.1 mM EDTA, 10 mM Tris-HCl, pH 8.0, 1 mM DTT, 0.5 % Triton) and dounced 10 times in a cold Dounce homogenizer. The resulting lysate was transferred to a new tube and mixed with 2 ml of sucrose buffer II (2 M sucrose, 5 mM magnesium acetate, 0.1 mM EDTA, 10 mM Tris-HCl, pH 8.0, 1 mM DTT). The sample was carefully layered on 2.2 ml of sucrose buffer II and was balanced using sucrose I buffer on top off the gradient, then centrifuged at 30,000 *g* for 45 min at 4°C (SW 40.1 rotor). After centrifugation, the supernatant (cytoplasmic fraction) was carefully removed and treated with proteinase K for 1h at 37°C, whereas the tight pellet consisting of nuclear fraction was dried at room temperature. Precipitation of the cytoplasmic fraction was performed using 0.1 volume of 3M sodium acetate, 2 µl paint pellet co-precipitant (Novagen) and 2 volumes of 100% ethanol, followed by 48h at −80°C. After centrifugation at 30,000g for 30 min at 4°C, washing steps with several volumes of 70% (v/v) ethanol were carried out, followed by a second centrifugation with identical conditions. The pellet was stored at −80°C for further RNA isolation. In parallel, the pellet of nuclei was gently rinsed with cold 1mM EDTA in PBS and resuspended with 200 µl of ice-cold glycerol storage buffer (50 mM Tris-HCl, pH 8.3, 40% (v/v) glycerol, 5mM MgCl_2_, 0.1mM EDTA), followed by RNA isolation or storage at −80°C.

RNA from the nuclear and cytoplasmic fractions was isolated using NucleoSpin RNAII (Macherey-Nagel) and eluted into 50 µl of water, following the manufacturer's protocol. Additional treatment with DNase-free RNase (Qiagen) was performed for 15 min at 22°C, to completely remove DNA, followed by a precipitation step as previously described. Quality and concentration of isolated RNAs were tested using RNA lab on chip (Agilent's Bioanalyzer 2100) and aliquots were reverse-transcribed with SuperScript™III (Invitrogen). QPCR was performed with intronic and exonic primers for selected genes in order to test DNA contamination in the samples lacking reverse transcriptase.

### cDNA synthesis

Four µg of pre-mRNA was used as template for cDNA synthesis. Reverse transcription was performed with 3 µg/µl random hexamers primers (Invitrogen) at 55°C for 1 h, using SuperScript™III first-strand Synthesis System (Invitrogen), according to the manufacturer's protocol. After the first strand was synthesized, a second-strand synthesis was generated by adding (5X) second strand synthesis buffer (Invitrogen), 25nM dNTPs, RNase H and DNA polymerase I (Invitrogen) for 2.5 h at 16°C. The double stranded cDNA was then cleaned up with the MinElute PCR purification kit (Qiagen) and eluted in 30 μl EB buffer.

### Custom library design

A customized probe library was generated using the eArray software (Agilent Technologies), as described in the user's guide. The synthetic 120-mer biotinylated oligonucleotide probes (baits) in solution were tiled along targeted intronic and exonic regions of the *DMD* and 3 different human control genes (*FXR1, CKLF* and *ACTB*). The genomic sequences corresponding to the 4 target genes were based on UCSC hg19-GRCh37 (chrX:31,137,336–33,357,726; chr3:180,630,234–180,695,106; chr16:66,586,466–66,600,190; chr7:55,70,372–55,66,779), respectively. To ensure capturing of intron containing pre-mRNA transcripts and low abundant transcripts, each sequence of the gene (except repeat areas) was covered generally by at least 4 baits, and the *DMD* promoter regions were covered on average by 8 baits. The maximum capacity of the synthesized library was up to 55K baits. The following parameters were chosen: sense strand, 1x capture-probe tiling frequency, layout strategy-centered, 20 bp overlap region between baits and avoid repeated masked regions.

### Pre- and post-hybridization sample preparations and Illumina sequencing

We created a library starting from 4 µg of pre-mRNA. Five cDNA capture libraries were generated from 3 different cultures cell lines: 7304, KM155 and 8220 cell lines. Our method has been slightly modified from the version provided by SureSelect^XT^ Target Enrichment System for Illumina Paired-End Sequencing Library (based on Agilent Technologies' updated versions). An additional cDNA synthesis step has been integrated to the original procedure Agilent prepped library protocol, which was designed for genomic DNA. Since our customized capture library is highly specific for 4 genes only, no rRNA depletion was done.

To define the best sample preparation method, we generated 2 different cDNA libraries using random primers and pre-mRNA isolated from nuclei of a differentiated healthy muscle cell line. In brief, cDNA, synthesized as previously described, was sheared using a Covaris instrument (Covaris, Inc.) at duty cycle 5, intensity level 5 and 200 cycles for burst (180s). The second method has been tested in parallel, in which pre-mRNA was fragmented before cDNA synthesis by the addition of 5 times fragmentation buffer (Ambion), heating at 70°C for 15 minutes and 5 minutes on ice. In both methods reverse transcription was applied to generate cDNA, using the same protocol as previously described and followed by purification with MinElute PCR purification kit (Qiagen). The cDNA ends were first repaired to obtain uniform double-stranded fragments with blunt ends, and then additional adenine residues were added to the fragment extremities to increase the efficiency of the following step. Finally, adaptors for Illumina sequencing were ligated in a concentration 2 fold less than provided in the instruction. Between each of the previous modification steps, a cleanup was performed using AMPure XP beads (Agencourt Bioscience Corporation) following the ratio beads/sample suggested by user's guide. With the exception after the adaptor ligation step, where the used ratio was 1:1 (volume). The following minor changes were made to the Agilent Technologies' protocol: the beads/sample thermo-mixture was incubated for 15 min at 22°C in a thermo mixer (1200 rpm), fresh 80% (v/v) ethanol was used and the elution step was performed at 37°C in a thermo-mixer.

A pre-hybridization amplification was performed with a limited number of cycles (5), reaching the required 500 ng of sample for the library hybridization step without amplification-induced biases. Primers were removed with 1 volume of AMPure XP beads, following previously described methods. This generated a cDNA library ranging in size from 160 to 660 bp. During the multiple steps of the sample preparations, the library quality was evaluated with an Agilent Bioanalyzer 2100 using HS DNA chips. The sample was concentrated to 3.4 μl and mixed with 2 µl of the customized capture library (Agilent Technologies, Inc. USA). The hybridization was further performed as described in the SureSelect^XT^ Target Enrichment System for Illumina Paired-End Sequencing Library manual. Post-hybridization amplification (12 cycles) with a different sequence index (barcode) per sample allowed pooling of samples, creating a multiplex libraries.

Amplified material was purified with AMPure XP beads, as early described previously. This step was repeated twice to minimize the amount of unused primers and reduce their sequence read bias. Using SureSelect^XT^ multiplex indexes, several post-capture amplified samples were pooled to a final concentration of 2 nM and with fragments size of 250–650 bp. The resulting pool of libraries was sequenced on an Hiseq PE 2*100 Illumina at a concentration of 7 pM. Output files in fastq format of the 5 Capture-pre-mRNA-seq containing paired-end reads and QC information were generated using CASAVA version 1.1.

### Analysis workflow

In order to detect non-sequentially spliced introns, exon blocks and to identify recursive splicing events, we used the pipeline described in Pulyakhina I. et al..^30^ For the classification and motif analysis scripts, we refer the reader to the https://git.lumc.nl/i.pulyakhina/pipeline_paper/tree/master. Non-processed fastq and bam files have been submitted to the European Nucleotide Archive under accession number http://www.ebi.ac.uk/ena/data/view/PRJEB9401.

### Alignment, post-alignment quality control

The pipeline first maped paired-end RNA-Seq data to a reference genome sequence (hg19, GRCh37) with GSNAP aligner^55^ (version 2012–07-12). Only uniquely mapped reads with a maximum of 5 mismatches in each end of a read are reported. All format conversions were done with SAMtools^56^ (version 0.1.18). For downstream analysis we extracted reads mapped to the target genes: *DMD, FXR1, ACTB* and *CKLF*. The annotations containing the coordinates of each exon and intron for each gene have been extracted from the RefSeq database (http://www.ncbi.nlm.nih.gov/refseq/). GRCh37.p13 RefSeq gene identifiers are NC_000023.10 (Chromosome X, *DMD*), NC_000003.11 (Chromosome 3, *FXR1*), NC_000016.9 (Chromosome 16, CKLF) and NC_000007.13 (Chromosome 7, *ACTB*).

To remove samples with low sequencing yields, we included only samples where the number of reads mapped to the *DMD* gene was > 500,000. Considering the length of the *DMD* transcript for Dp427m (2,092,329 nt) and the length of paired-end reads (2 times 100 nt) this cut-off means that each position of the *DMD* gene was covered on average around 50 times. The same cut-off was applied to RNA and DNA samples.

### Coverage: median coverage of exons and introns

The median value of the coverage of each position within the corresponding intron or exon was calculated using the “median ()” command in R (version 2.5.1.1). We only included areas of introns and exons that were covered by our designed probes. We excluded such regions as promoters, UTRs and pseudogenes that can potentially influence the coverage and bias the median coverage of introns (Table S2). This calculation reflected an accurate coverage for all introns, except intron 40, where a small area was highly covered even after removal of a known UTR. We considered this intron an outlier. Positions with zero coverage were also included in calculating the median. To make the coverage comparable in the different cell line samples, the coverage for each exon and intron was normalized using the average coverage of the *DMD* exons in all cell lines, and the median of the normalized coverage was used for further analysis. Correlation of the median of the intron coverage between different samples was calculated using Pearson's correlation and reported as R-values throughout the paper.

### Coverage: no GC bias

To estimate the influence of GC content on the median coverage, we calculated the length of each exon and intron and the fraction of nucleotides that consisted of “G” and “C” and built a linear regression of the coverage and the GC content for both DNA and RNA samples. Since no significant correlation of GC content with median coverage of introns was found (*P*-value 0.26 or higher), median coverage values were not normalized for the GC content.

### Classification of reads

Reads were classified in 3 categories, based on the location of the alignment and the distance between the 2 mapped ends of a read pair (expected insert size is approximately 400 nt). According to the reference alignment the reads were aligned in the exon, intron, exon-intron boundary or exon-exon junction. Following the reads were classified as pre-, intermediate- and post-splicing. Mainly, reads belong to the intermediate-splicing category were used for downstream analysis. If the distance between the 2 mapped ends of a read pair were higher or lower than the expected insert size, reads were labeled as “large” or “normal”, respectively. Reads fully mapped to an exon were classified into a separate category.

### Splicing order analysis

The median coverage of each intron was used to extrapolate an estimated the order of intron removal, based on an assumed correlation between the coverage depth and the relative speed of intron removal: the slower the intron is removed, the longer the target is available and the higher is the coverage. The average of the median normalized intron coverage from the 5 (Capture-pre-mRNA-seq) libraries was used for downstream analysis. We assessed 26 units of 5 introns, shifting 3 introns for each subsequent unit. Next we compared the normalized coverage of each intron in the unit, defining the cut-offs values of 90 and 130. Cut offs were chosen such that 2 equal bins were created. These bins were clearly delineated with only a few introns not fitting in either category. These introns were placed in the intermediate category. Introns with an average coverage of less than 90 (low coverage) were considered to be spliced quickly (fast splicing), while introns with an average coverage of more than 130 (high coverage) were considered to be spliced slowly (slow splicing).

A paired-end split reads-based method was applied for a straightforward analysis to confirm the results of the coverage-based analysis. We counted paired-end reads having one split read spanning an exon-exon (ex-ex) junction and the second read mapped to the intron (in) immediately up- or downstream (Fig. S3A). The identification of this type of fragments is limited by the size of the captured fragments (250–650nt). However, internal *DMD* exons range in size from 32–275nt, and this allows for the detection of splicing intermediates that involve 2 or more consecutive exon-exon junctions. We used the total number of these split reads in all our 5 libraries, and calculated the splice-ratio for each intron as follows:

$$\mathrm{Splice-ratio}=\frac{\text{S}}{\text{S}+\text{NS}}=\frac{\left(\text{e}\text{x}(\text{n})-\text{e}\text{x}(\text{n}+1)∼\text{i}\text{n}(\text{n}+1)\right)}{\left[(\text{e}\text{x}(\text{n})-\text{e}\text{x}(\text{n}+1)∼\text{i}\text{n}(\text{n}+1))+(\text{i}\text{n}(\text{n})∼\text{e}\text{x}(\text{n}+1)-\text{e}\text{x}(\text{n}+2))\right]}$$

In this formula, (in_(n)_∼ex_(n+1)_–ex_(n+2)_) or (S) reflects the number of read pairs supporting sequential splicing, where one read of a paired-end spans an exon-exon junction arising from the splicing of the intron, while the other maps to the intron immediately downstream (e.g., for intron 33 this would be the number of read pairs where one read spans the exon 33–34 junction and the other read maps to the intron 34).

(in_(n)_∼ex_(n+1)_-ex_(n+2)_) or (NS) reflects the number of read pairs supporting non-sequential splicing, where one read of a paired-end maps to an intron, while the other covers the exon-exon junction resulting from the splicing of the intron immediately downstream (e.g., for intron 33, one read pair would map to intron 33, while the other would map to the exon 34–35 junction).

We calculated the splice-ratio for each intron and defined introns with a splice-ratio between 0.5 and 1 as sequentially spliced, as the result of reads supporting sequential splicing (S) are more than non-sequential (NS), while introns with a splice ratio of <0.5 were defined as non-sequentially spliced.

### Recursive and nested splicing

Potential recursive and nested splicing events were predicted using split reads belonging to the intermediate-splicing category. The first and second reads of each read pair were analyzed separately as single end reads. Each uniquely mapped read that contained a gapped alignment was selected. Two base pairs at the beginning and at the end of the gap, that were not covered by the reads, were classified as candidate donor and acceptor splice sites. The splice sites were assigned based on the split point of the read, the alignment of the flanking sequences and considering the 2 nucleotides that had the highest similarity to the splice site sequence. Identified split reads containing 2 annotated splice sites were discarded. However, when the donor and/or the acceptor were not present in the reference annotation of the gene, the read was selected for downstream analysis (**see** Fig. 6). We added the number of reads of the same cell line and performed the analysis for the 3 different cell lines and selected events present in all 3 datasets.

Predicted recursive splicing events were reported as a matrix containing the upstream and downstream genomic nucleotides flanking the position where the read was split. A matrix where the rows contained positions of the donor and the columns contained positions of the acceptor splice sites was created, and the intersecting cell represented the number of reads for that specific pair of the donor and acceptor site. All the splicing events were also listed in a separate text (Table S4). We analyzed events happening within one intron. Reads split within the first 50 intronic nucleotides (near the exon-intron boundary) were not considered, as they were thought to represent variation in normal exon-exon splicing (including the well established NAGNAG splice site variations). We classified the events based on Fig. 1, as recursive (5′RS, 3′RS) and nested splicing.

### Motif analysis

We performed motif analysis on the donor and acceptor splice sites from the predicted recursive and nested splicing events. We extrapolated the sequence of the annotated and non-annotated 5′ and 3′splice sites from each event and additionally the 2 nucleotides upstream from each donor splice site and 2 positions downstream from the acceptor splice site. Four extracted nucleotides were used to create the sequence logo using Weblogo software (http://weblogo.berkeley.edu/).

### Experimental validation

For exon block validation, experiments were performed on nuclear RNA from 2 cell lines and triples were performed independently 3 times. For recursive splicing validation, experiments were performed on nuclear and cytoplasmic RNA from 2 cell lines. PCR primers for all targeted *DMD* introns and exons were designed using Genomic refseq ID NG 012232.1 (Table S6). As a template, 1 µg of isolated pre-mRNA was reverse-transcribed using SuperScript III (Invitrogen), following the manufacturer's instructions.

Exon blocks were validated using qPCR. Quantitative RT-PCR was performed in a 8 µl reaction containing 4 µl SYBR Green master mix (ThermoScientific), 0.2 pM each primer, and 2 µl of diluted cDNA template. PCR was performed on the LightCycler 480 (Roche Diagnostics Ltd.). Thermal Cycling conditions were as follows: 50°C for 2 min, 95°C for 10 min, 45 cycles of 95°C for 15 s and 60°C for 1 min. Analysis of the raw data and PCR efficiency was performed using the LinRegPCR software.^57^ For all combinations of primers a Reverse Transcriptase negative control sample was included to exclude DNA contamination. A pair of primers covering the unspliced intron and immediate downstream exon was used to confirm the ability of the intronic primer to generate a PCR fragment. For primers spanning an exon-exon junction, there was little flexibility for primer design, resulting sometimes in low primer efficiencies. For each exon block, all qPCRs were performed simultaneously. *HPRT* was used as a reference gene.

PCR followed by Sanger sequence was used to confirm the specific splice junctions in the predicted exon blocks and splicing events. For the exon blocks, we designed primers in the unspliced intron and in the last exon, where for the splicing events, we used specific primers upstream and downstream the split reads (Table S3). cDNA was generated from 100 ng of pre-mRNA using 2x master mix buffer (Ambion) and 1 µl of enzyme in a total volume of 50 µl. PCR reactions were carried out as per manufacturer's instructions. Each assay was performed for the 2 different cell lines. The PCR was performed using initial denaturation at 98°C for 2 min followed by 35 cycles of (98°C for 15s, 55°C for 30s, 72°C for 30s) and a final extension of 72°C for 10min. The PCR products were subsequently analyzed on a 2% agarose gel. After purification with the MinElute PCR purification kit (Qiagen), the amplicons were analyzed using Sanger sequencing. The results were blasted in the UCSC genome browser to confirm the correct sequence and identify intron-exons and exon-exon junctions for each exon block.

## Supplementary Material

KRNB_A_1125074_Supplementary_Figure_1-3_and_Supplementary_Table_1-4.pdf
