# Supplementary material for: Non-sequential and multi-step splicing of the dystrophin transcript
Source: RNA Biol. 2015 Dec 15;13(3):290–305. doi: 10.1080/15476286.2015.1125074 (PMC4829307; doi:10.1080/15476286.2015.1125074)
Supplement: KRNB_A_1125074_Supplementary_Figure_1-3_and_Supplementary_Table_1-4.pdf [file krnb-13-03-1125074-s001.pdf]

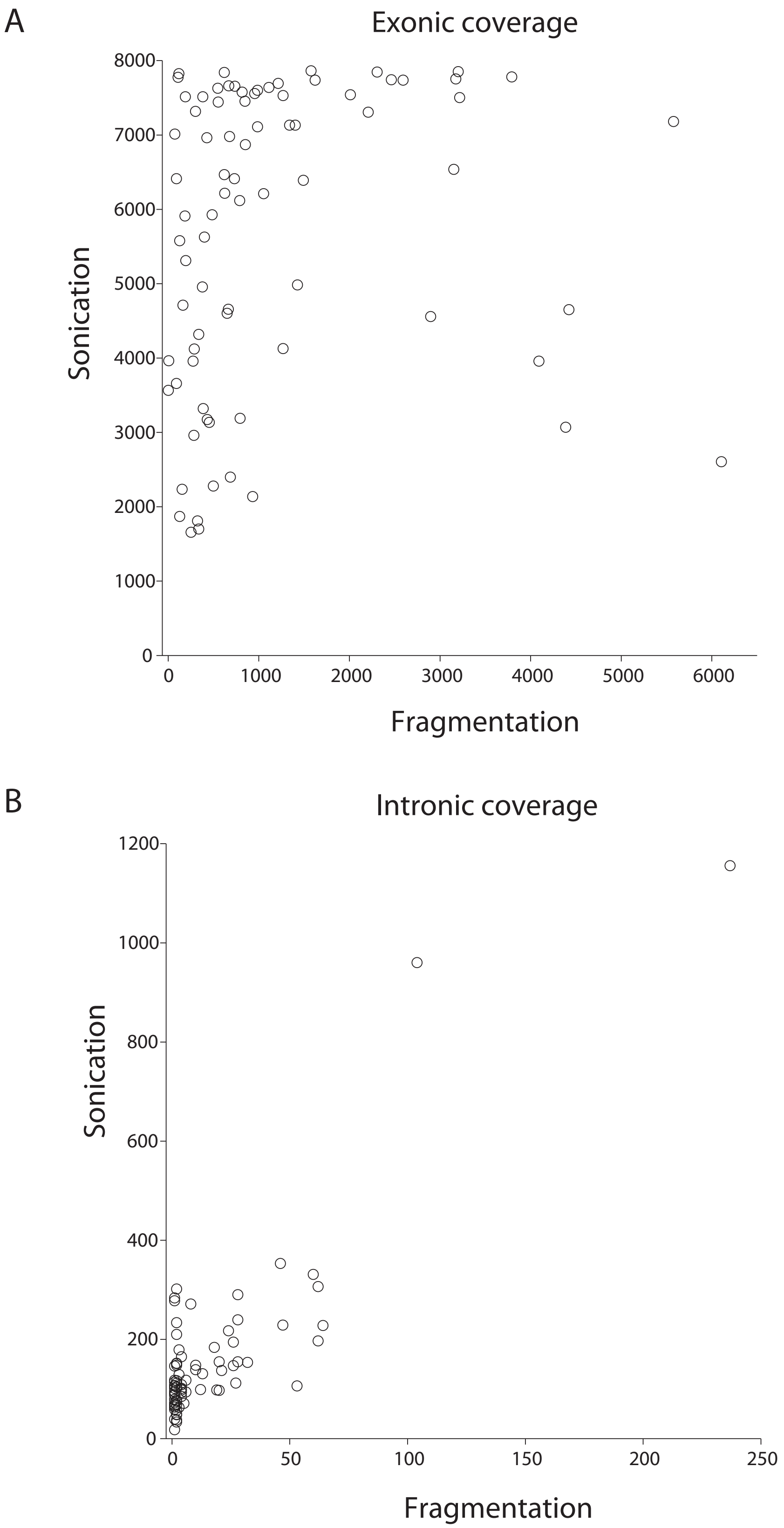


**Figure S1**. Comparison of two sample preparation methods: fragmentation versus sonication. Scatter plot representing exonic and intronic DMD coverage as a result of sonication or fragmentation methods. **(A)** Exon coverage. **(B)** Intron coverage.

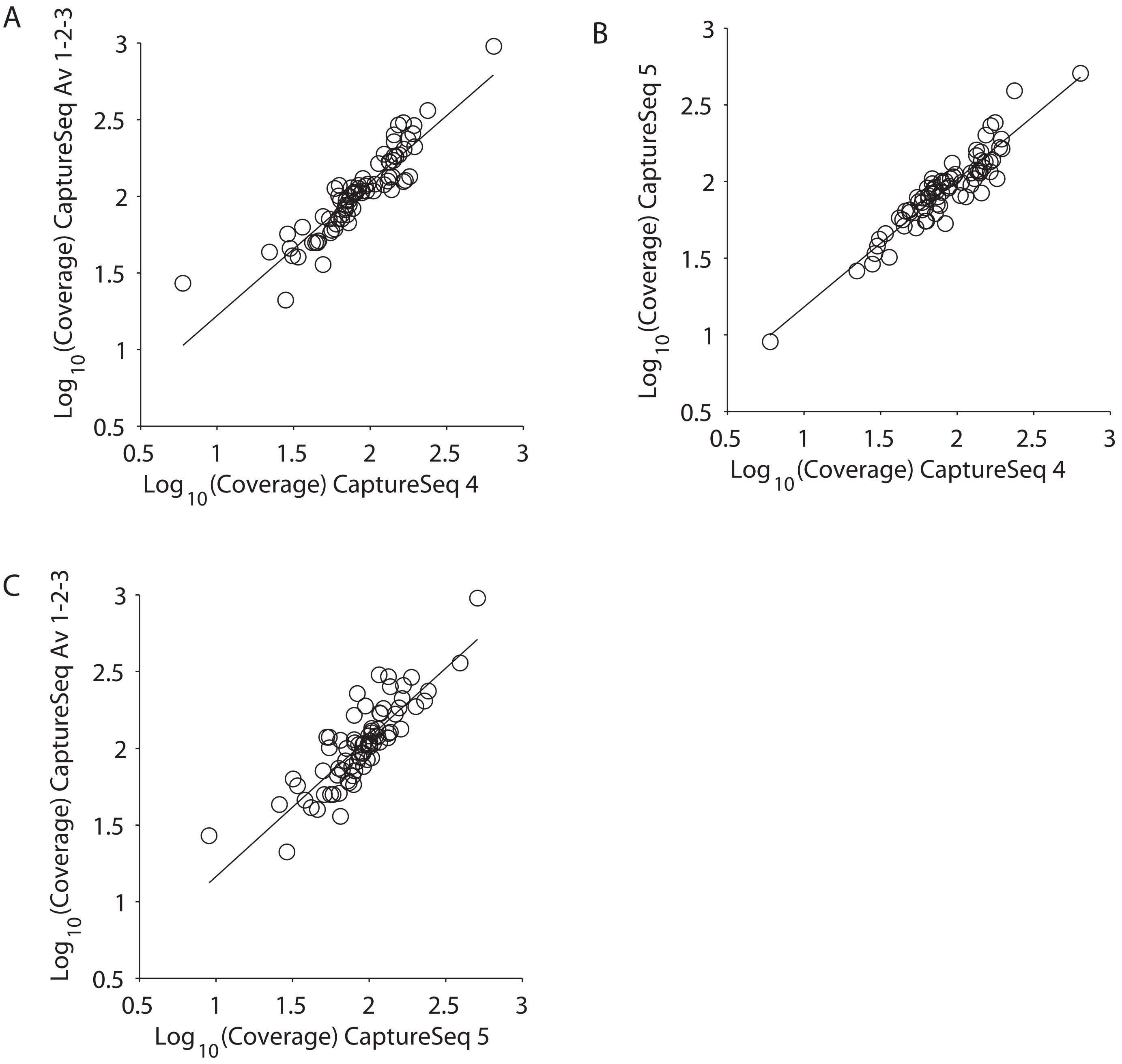


**Figure S2. Scatter plot and regression line showing high correlation of the intron coverage between different cell lines. (A)** CaptureSeq 1, 2, 3 (average of the median normalized intron coverage) was plotted against CaptureSeq 4 (R= 0.91, P= 4.5. 10-31). **(B)** CaptureSeq 5 was plotted against CaptureSeq 4 (R= 0.94, P= 2. 10-35). **(C)** CaptureSeq 1, 2,3 was plotted against CaptureSeq 5 ( R= 0.84, P= 1.8. 10-22). Representations of the data points have been provided using logarithmic scales.

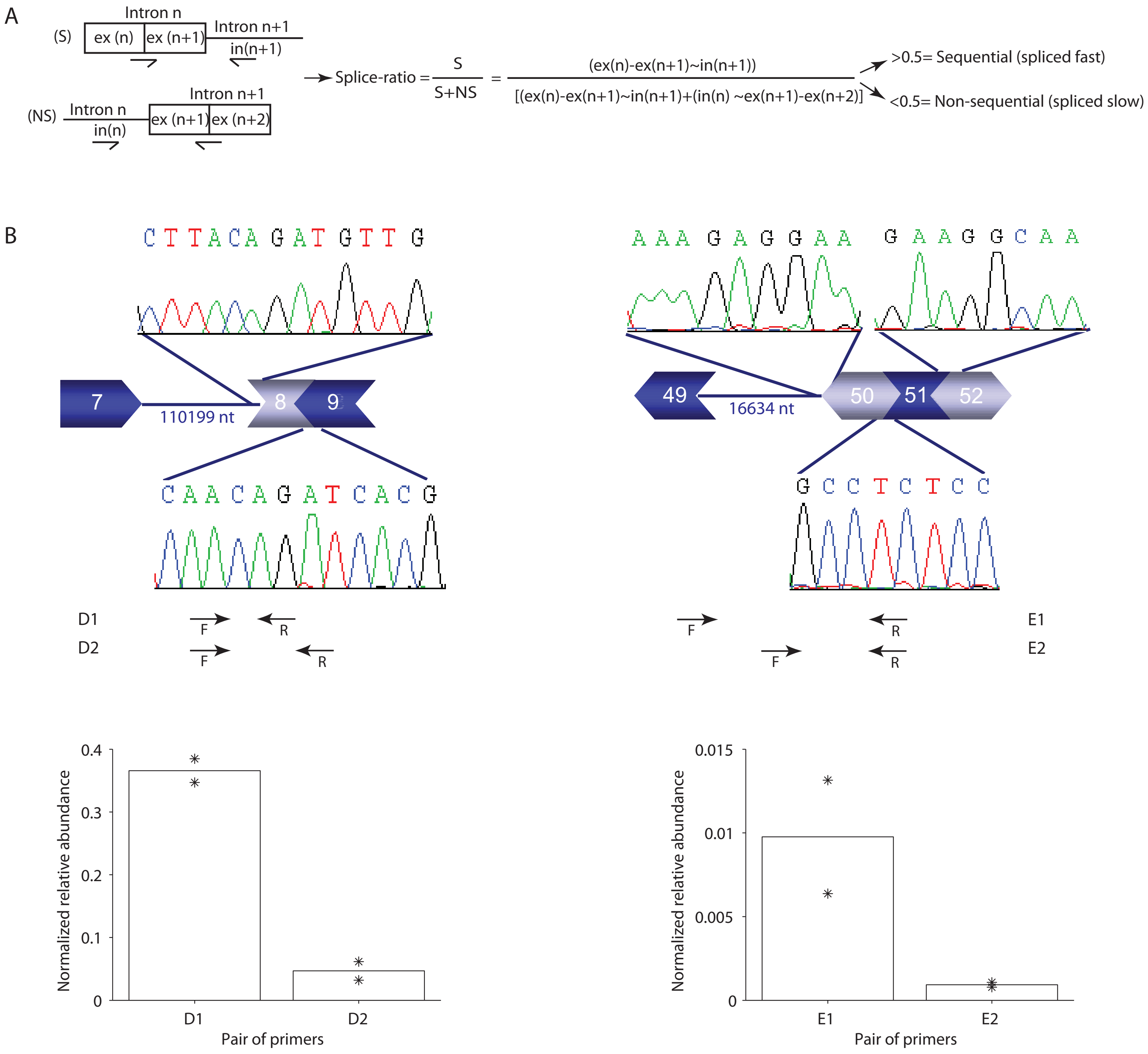


**Figure S3. Schematic illustration of the splice-ratio used in the paired-end reads analysis for the identification and the validation of the exon blocks.** (**A)** In the two left cartoons, the horizontal arrows indicate reads spanning exon-exon junction or fully aligned to the intron. On the right side, example of splice-ratio formula used to calculate splicing stage for intron (n) is shown. In the numerator, the sequential splicing (S) supported by paired-ends reads (ex(n)-ex(n+1)~in(n+1)) is represented in the upper left drawing. In the denominator, the sum of number of reads indicating sequential (S) and non-sequential (NS) is represented as [ (ex(n)-ex(n+1)~in(n+1))+ (in(n) ~ex(n+1)-ex(n+2))] in the upper and lower left cartoons. **(B)** Experimental validation of the predicted non-sequential splicing of intron 7 and 49. Eletropherograms of Sanger sequencing show intron/exon and exon/exon junctions of the non-sequential and sequential splicing, respectively, PCR and sequencing analysis have been performed using forward and reverse primers in intron 7 and exon 9, and in intron 49 and exon 52 (upper panel). Schematic representation of qRT-PCR showing unspliced and spliced introns of the predicted exon blocks, and relative location of the primers used (lower panel).

**Table S1**. **Overview of the analysis of six Capture-seq libraries using three different human muscle cell lines**

| Libraries | Cell lines | Material | Diff. days | Hybri cycles | Total number of reads | Reads mapped to DMD | % reads mapped to DMD | Reads mapped to DMD introns | % intronic reads mapped to DMD | Reads mapped to DMD exons | % exonic reads mapped to DMD | % pre-splicing | % inter. splicing | % post-splicing |
|---|---|---|---|---|---|---|---|---|---|---|---|---|---|---|
| CaptureSeq 1 | 7304 | pre-mRNA | 8 days | 17 | 10,780,572 | 749,012 | 6.95 | 533,917 | 71.28 | 180,612 | 24.11 | 79.21 | 3.89 | 16.89 |
| CaptureSeq 2 | 7304 | pre-mRNA | 8 days | 17 | 11,049,803 | 898,525 | 8.13 | 605,796 | 67.42 | 270,668 | 30.12 | 74.18 | 3.68 | 22.14 |
| CaptureSeq 3 | 7304 | pre-mRNA | 14 days | 17 | 8,589,435 | 2,507,322 | 29.19 | 2,057,934 | 82.08 | 336,801 | 13.43 | 88.99 | 1.54 | 9.47 |
| CaptureSeq 4 | KM155 | pre-mRNA | 8 days | 17 | 9,490,257 | 4,017,992 | 42.34 | 3,078,322 | 76.61 | 943,852 | 23.49 | 80.68 | 2.64 | 16.68 |
| CaptureSeq 5 | 8220 | pre-mRNA | 8 days | 17 | 9,326,334 | 6,140,259 | 65.84 | 4,819,545 | 78.49 | 1,176,916 | 19.17 | 83.69 | 2.07 | 14.23 |
| CaptureSeq 6 | 7304 | DNA | 8 days | 21 | 11,564,676 | 6,572,037 | 56.83 | 5,884,255 | 89.53 | 63,068 | 0.96 | 99.88 | 0.12 | 0.00 |

**Table S2. Pseudogenes, promoters, UTRs and micro RNA sequences in intragenic *DMD* region.**

| Intron | Genomic position | Type |
|---|---|---|
| Intron 1 | ChrX:33,357,077-33,358,066 | DP427L |
| Intron 1 | ChrX:33,229,431-33,229,677 | DP427m |
| Intron 1 | ChrX:33,146,283-33,146,546 | DP427p |
| Intron 1 | ChrX:33,059,512-33,059,838 | TBCAP1 (Retro-TBCA) |
| Intron 3 | ChrX:32,866,024-32,866,122 | snoU13 |
| Intron 7 | ChrX:32,773,057-32,774,517 | DMD-AS3 |
| Intron 11 | ChrX:32,659,591-32,659,676 | MIR548F5 |
| Intron 13 | ChrX:32,601,773-32,601,869 | MIR3915 |
| Intron 18 | ChrX:32,534,714-32,536,124 | UTR |
| Intron 29 | ChrX:32,430,175-32,430,372 | DP260 |
| Intron 40 | ChrX:32,360,748-32,360,969 | UTR |
| Intron 44 | ChrX:32,173,486-32,173,586 | DP140 |
| Intron 44 | ChrX:32,223,913-32,225,169 | NPM1P8 |
| Intron 55 | ChrX:31,526,355-31,526,600 | DP116 |
| Intron 62 | ChrX:31,284,947-31,285,070 | DP71 |
| Intron 62 | ChrX:31,279,214-31,279,847 | DMD-AS2 anitsense |
| Intron 70 | ChrX:31,196,312-31,196,783 | UTR of DP40 |

**Table S3. List of pairs of primers used in the analysis.**

| Primer | Sequence | Experimental validation |
|---|---|---|
| Intron 7F | AACTTTGATTTGTTCATTATCCTTTT | Exon block |
| Exon 8R | ACTTGTTGAGGCAAAACTTGGA | Exon block |
| Exon 8-9R | CTGACCGTGATCTGTTGAGAAT | Exon block |
| Exon 9R | GAAGTTCTCTCATATCCCTGTGC | Exon block |
| Exon 13F | GAACAAGAACAAGTCAGGGTCA | Exon block |
| Intron 13F | TGGCAAATTATTCATGCCATT | Exon block |
| Exon 14R | CGTTGCCATTTGAGAAGGAT | Exon block |
| Exon 14/15R | CACTAAAAAGGCACTGTTCTTCAGTAA | Exon block |
| Exon 15/16R | CGCTTTTAAAACGGCCAGTTTTTG | Exon block |
| Intron 16R2 | CAACACCGGGCAAAGTTATC | Exon block |
| Exon 33/34F | CTG GGAGCAAAGGTAACAGAAAGA | Exon block |
| Intron 34R | CATGGTCCTGAAAAGCACAG | Exon block |
| Intron 34F3 | TCATAGTTACCCAACAATGAAGC | Exon block |
| Exon 35R | GGGAGGTGACAGCTATCCAG | Exon block |
| Intron 35F | GTATATGCTGGCTCTTAATTCAA | Exon block |
| Exon 35/36F | AATCTTTTGTTGGAATACCAGAAAC | Exon block |
| Exon 35/36R | GTTTCTGGTATTCCAACAAAAGAT T | Exon block |
| Exon 36/37F | ACGTGCTTAAGCGTTTAAAGGC | Exon block |
| Exon 36/37R2 | CTGCCTTTAAACGCTTAAGCACGTCT | Exon block |
| Exon 37R | GCTCTGAGATTTGGGGCTCT | Exon block |
| Intron 37R | TTGGCATTCATTTTCCTTTTG | Exon block |
| Exon 44F | CCTGAGAATTGGGAACATGC | Exon block |
| Intron 44F | CATGGGGCTTCATTTTTGTT | Exon block |
| Exon 45R | AGCTGTTTGCAGACCTCCTG | Exon block |
| Intron 45F | GCCATGTTTGTGTCCCAGTT | Exon block |
| Exon 45-46R | GTTCTTCTAGCCTCTTTTTTCTC | Exon block |
| Intron 46F | CTCGGTCAAGTCGCTTCATT | Exon block |
| Eoxn 46/47F | GAGCAAGTCAAGTTACTGGTGG | Exon block |
| Exon 46/47R | CCACCAGTAACTTGACTTGCTC | Exon block |
| Intron 47F | TGCTGCTAAAATAACACAAATCA | Exon block |
| Exon 47/48R | GCTCTGGAAACCTTTATCCACT | Exon block |
| Exon 48/49R | TATTTCAGTTTCCTGAACGTCAAA | Exon block |
| Exon 49F | AACAACCGGATGTGGAAGAG | Exon block |
| Intron 49R | TGGCCAGTATTTCCTTACAAGTT | Exon block |
| Intron 49F | TTCACCAAATGGATTAAGATGTTC | Exon block |
| Exon 50-51R | TCTGAGTAGGAGAGGCTCCAATAG | Exon block |
| Exon 52R2 | TTCTTCCAACTGGGGACGC | Exon block |
| Intron 65F | AGAGTTCACACATCATTGAGCA | Exon block |
| Intron 67R | AACGAAGCTCTGTGGGTTTTT | Exon block |
| Exon 53F | TTCAGAATCAGTGGGATGAAG | Recursive 5'RS |
| Exon 53F2 | GTCTTAGGACAGGCCAGAGC | Recursive 5'RS |
| Inttron 53R | TTCACATTCTAGTACCACATTGACTT | Recursive 5'RS |
| Exon 43F | GACATTATTCATAGCAAGAAG | Recursive 5'RS |
| Intron 43AR | CTTATTTTACTGGCTGGTTGTG | Recursive 5'RS |
| Intron 4F | CTATCTTCCTGCTTACACAGTG | Recursive 3'RS |
| Intron 4F2 | GGTTACTGGAATCTAAGTTCCT | Recursive 3'RS |

| | | |
|---|---|---|
| Exon 5R | CCAGTGGAGGATTATATTCC | Recursive 3'RS |
| Exon 5R2 | CAAGAGTCAGTTTATGATTTC | Recursive 3'RS |
| Exon 7AF | AGTCAGCCACACAACGACTG | Recursive intermezzo |
| Intron 7AF | GGCTTTCGTATCAGGATGATG | Recursive intermezzo |
| Intron 7AR | CAACCAAAAAGAGTCCAGGA | Recursive intermezzo |
| Exon 8R | ACTTGTTGAGGCAAAACTTGGA | Recursive intermezzo |
| Exon 33F | GTGGAAATGGTGATAAAGACTGG | Recursive intermezzo |
| Intron 33AF | GTCTCATGACCAGCTCCACT | Recursive intermezzo |
| Intron 33AR | TTTGAGTTGAGCGAAGTGAG | Recursive intermezzo |
| Exon 34R | TTTCCTTTCGCATCTTACGG | Recursive intermezzo |
| Exon 34F | GATCAGCAGTTGAAGGAATGC | Recursive intermezzo |
| Intron 34AF | GATTTCGAGTGGACCAGGAC | Recursive intermezzo |
| Intron 34AR | AAGTAGTCCTGTGGCTGTCT | Recursive intermezzo |
| Intron 34BF | AGAAAGAATGGGAGAACTTCC | Recursive intermezzo |
| Intron 34BR | CTGCAGCTGTCCCTTCAGTT | Recursive intermezzo |
| Exon 35R | GGGAGGTGACAGCTATCCAG | Recursive intermezzo |
| Intron 43F-L1 | TTGCTTGCTATCGTCACTGC | Nested |
| Inttron 43R-L1 | CCTCAAAGGAATTCTAGTTTTTATAGG | Nested |
| Intron 43L1-F2 | CTTGCTGTGTGTCGCTCATT | Nested |
| Intron 43L1-R2 | TGTATGGACACTGCCTTATGAAA | Nested |

**Table S4. Predicted recursive and nested splicing in *DMD*. Genomic positions (NC_000023.10), size of the spliced sequence and splice site motifs are reported for each event.** (Experimentally validated events in bold)

| DP427m | Genome positions of split read | Event | Gap distance | Donor | Acceptor |
|---|---|---|---|---|---|
| Intron1 | chrX:33227925-33229399 | 5'RS | 1473 | TTgt | agAT |
| Intron1 | chrX:33226732-33227887 | nested | 1154 | AGgt | agAT |
| Intron1 | chrX:33222166-33222236 | nested | 69 | TGgc | tgAT |
| Intron1 | chrX:33220779-33221205 | nested | 425 | ACct | acAG |
| Intron1 | chrX:33214128-33214338 | nested | 209 | TTgt | atAT |
| Intron1 | chrX:33214114-33222808 | nested | 8693 | AGgt | agCT |
| Intron1 | chrX:33213332-33215241 | nested | 1908 | AGgt | agAG |
| Intron1 | chrX:33213298-33215241 | nested | 1942 | AGgt | agCT |
| Intron1 | chrX:33211797-33214016 | nested | 1473 | AGgt | agGT |
| Intron1 | chrX:33211192-33219312 | nested | 2218 | CAct | acCT |
| Intron1 | chrX:33210975-33219269 | nested | 8293 | TTcc | cgCA |
| Intron1 | chrX:33210974-33219268 | nested | 8293 | TCca | gcAA |
| Intron1 | chrX:33210650-33219279 | nested | 8628 | AGgt | agGC |
| Intron1 | chrX:33208588-33208913 | nested | 324 | AAga | aaGA |
| Intron1 | chrX:33208581-33208906 | nested | 324 | ATca | atCA |
| Intron1 | chrX:33204601-33206444 | nested | 1842 | AAgt | agCA |
| Intron1 | chrX:33202683-33210910 | nested | 8226 | GGgt | agGA |
| Intron1 | chrX:33197575-33198252 | nested | 676 | CGgc | cgGC |
| Intron1 | chrX:33197569-33209810 | nested | 12240 | CCca | ccCA |
| Intron1 | chrX:33197569-33198222 | nested | 652 | CCca | ccCA |
| Intron1 | chrX:33197567-33198220 | nested | 652 | CAgc | caAA |
| Intron1 | chrX:33197190-33212586 | nested | 15395 | ATga | ctGA |
| Intron1 | chrX:33197056-33198202 | nested | 1145 | AGgt | agGG |
| Intron1 | chrX:33194811-33209810 | nested | 14998 | CCca | ccCA |
| Intron1 | chrX:33194806-33209796 | nested | 14989 | AGgc | agTG |
| Intron1 | chrX:33193754-33221215 | nested | 27460 | TGgt | agTC |
| Intron1 | chrX:33192789-33207271 | nested | 14481 | TTct | ttGT |
| Intron1 | chrX:33192770-33207271 | nested | 1450 | TTct | atTT |
| Intron1 | chrX:33192721-33192814 | nested | 92 | AAcc | atCC |
| Intron1 | chrX:33192669-33207271 | nested | 14601 | TTct | ttGT |
| Intron1 | chrX:33192104-33207271 | nested | 15166 | TTct | atGT |
| Intron1 | chrX:33190840-33190935 | nested | 94 | ATta | acTG |
| Intron1 | chrX:33190805-33192302 | nested | 1496 | AGgt | agGA |
| Intron1 | chrX:33188414-33192459 | nested | 4044 | GGtt | ttTC |
| Intron1 | chrX:33186383-33198544 | nested | 12160 | GTgt | agTA |
| Intron1 | chrX:33186356-33198544 | nested | 12187 | GTgt | ttGT |
| Intron1 | chrX:33186350-33198554 | nested | 12203 | GTgc | atAT |
| Intron1 | chrX:33185249-33201462 | nested | 16212 | AGgt | agAA |
| Intron1 | chrX:33185249-33186870 | nested | 1620 | AGgt | agAA |

| | | | | | |
|---|---|---|---|---|---|
| Intron1 | chrX:33184588-33185408 | nested | 819 | ACct | acTG |
| Intron1 | chrX:33181387-33205434 | nested | 24046 | CCct | gaGC |
| Intron1 | chrX:33181183-33203088 | nested | 21904 | AGgt | tgGC |
| Intron1 | chrX:33181132-33208851 | nested | 27718 | GCgg | agAG |
| Intron1 | chrX:33180205-33180527 | nested | 321 | TCct | acTC |
| Intron1 | chrX:33180189-33180527 | nested | 337 | TCct | tcTA |
| Intron1 | chrX:33180098-33192457 | nested | 12358 | TTtt | agAA |
| Intron1 | chrX:33179970-33188404 | nested | 8433 | TTca | agTA |
| Intron1 | chrX:33179966-33192459 | nested | 12492 | GGtt | atTT |
| Intron1 | chrX:33179964-33192457 | nested | 12492 | TTtt | ttAA |
| Intron1 | chrX:33177714-33229399 | 5'RS | 51684 | TTgt | agGA |
| Intron1 | chrX:33177049-33177138 | nested | 88 | ATaa | tgAG |
| Intron1 | chrX:33175828-33209807 | nested | 33978 | AAca | aaAG |
| Intron1 | chrX:33173772-33185916 | nested | 12143 | TGgg | agGC |
| Intron1 | chrX:33173710-33181037 | nested | 7326 | ACct | acCT |
| Intron1 | chrX:33165852-33178810 | nested | 12957 | AGgt | agGA |
| Intron1 | chrX:33163059-33170186 | nested | 7126 | TAca | aaTC |
| Intron1 | chrX:33162818-33201226 | nested | 38407 | TAgt | acTC |
| Intron1 | chrX:33162647-33162724 | nested | 76 | TGat | tgAT |
| Intron1 | chrX:33161066-33183123 | nested | 22056 | TCct | tcCT |
| Intron1 | chrX:33159888-33171492 | nested | 11603 | TGca | agCA |
| Intron1 | chrX:33159641-33176060 | nested | 16418 | ACtc | acTC |
| Intron1 | chrX:33159606-33162809 | nested | 3202 | TCct | gcAC |
| Intron1 | chrX:33159412-33229399 | 5'RS | 69986 | TTgt | agCA |
| Intron1 | chrX:33157077-33168642 | nested | 11564 | AGgt | agGA |
| Intron1 | chrX:33157038-33170939 | nested | 13900 | GTgt | agGA |
| Intron1 | chrX:33156928-33168624 | nested | 11695 | CAac | agAA |
| Intron1 | chrX:33156927-33195715 | nested | 38787 | AAcc | gaAG |
| Intron1 | chrX:33152291-33187469 | nested | 35177 | GGct | agGA |
| Intron1 | chrX:33151965-33158123 | nested | 6157 | AAcc | taTC |
| Intron1 | chrX:33151962-33158122 | nested | 6159 | ACca | ccAA |
| Intron1 | chrX:33151904-33159108 | nested | 7203 | ATct | acCA |
| Intron1 | chrX:33151300-33156429 | nested | 5128 | TCct | acCT |
| Intron1 | chrX:33148004-33172809 | nested | 24804 | GCct | acCC |
| Intron1 | chrX:33146311-33150379 | nested | 4067 | AAgt | agGT |
| Intron1 | chrX:33143925-33170382 | nested | 26456 | ACct | atCT |
| Intron1 | chrX:33143138-33229399 | 5'RS | 86260 | TTgt | agAT |
| Intron1 | chrX:33142898-33170558 | nested | 27659 | CAgc | caAA |
| Intron1 | chrX:33140067-33165198 | nested | 25130 | TGgg | tgGC |
| Intron1 | chrX:33137281-33181639 | nested | 44357 | CAct | acAA |
| Intron1 | chrX:33137212-33137381 | nested | 168 | ATgt | gtAG |
| Intron1 | chrX:33137163-33141766 | nested | 4602 | AGgt | agTG |
| Intron1 | chrX:33135934-33163091 | nested | 27156 | TGgt | agTT |
| Intron1 | chrX:33133716-33136316 | nested | 2599 | AGgc | agGC |
| Intron1 | chrX:33133708-33134276 | nested | 567 | AGtg | ctTG |

| | | | | | |
|---|---|---|---|---|---|
| Intron1 | chrX:33133705-33134273 | nested | 567 | GCag | gcAG |
| Intron1 | chrX:33133701-33134269 | nested | 567 | TGgc | tgAG |
| Intron1 | chrX:33133581-33140301 | nested | 6719 | TGgt | agGC |
| Intron1 | chrX:33130944-33131473 | nested | 528 | AGgc | agGC |
| Intron1 | chrX:33129378-33143113 | nested | 13734 | GGct | gcCT |
| Intron1 | chrX:33129358-33129455 | nested | 96 | AAat | aaAT |
| Intron1 | chrX:33128043-33128483 | nested | 439 | AGtt | tgTT |
| Intron1 | chrX:33124892-33145966 | nested | 21073 | AGgt | agCC |
| Intron1 | chrX:33118363-33131452 | nested | 13088 | GCct | caTT |
| Intron1 | chrX:33117935-33123485 | nested | 5549 | ACct | acAG |
| Intron1 | chrX:33112615-33169604 | nested | 56988 | TTga | atGT |
| Intron1 | chrX:33110008-33124045 | nested | 14036 | TGgt | ggAA |
| Intron1 | chrX:33106705-33107319 | nested | 613 | GTcc | gcCT |
| Intron1 | chrX:33098391-33105448 | nested | 7056 | ATtt | ggTA |
| Intron1 | chrX:33096464-33229399 | 5'RS | 132934 | TTgt | agGA |
| Intron1 | chrX:33091050-33114917 | nested | 23866 | AGgt | agAT |
| Intron1 | chrX:33089317-33106449 | nested | 17131 | GTgg | tgGG |
| Intron1 | chrX:33086570-33086636 | nested | 65 | ATtt | atAA |
| Intron1 | chrX:33085962-33088750 | nested | 2787 | AGat | ttTG |
| Intron1 | chrX:33084094-33099622 | nested | 15527 | AGgt | agGA |
| Intron1 | chrX:33082224-33120718 | nested | 38493 | TCat | aaGT |
| Intron1 | chrX:33080549-33106507 | nested | 25957 | CAgt | agGA |
| Intron1 | chrX:33077752-33101154 | nested | 23401 | CTgc | atAT |
| Intron1 | chrX:33077671-33101162 | nested | 23490 | CTct | agCT |
| Intron1 | chrX:33071450-33111290 | nested | 39839 | GGct | gcAT |
| Intron1 | chrX:33070351-33091855 | nested | 21503 | CTgc | agAT |
| Intron1 | chrX:33068788-33090494 | nested | 21705 | TGgt | tgTG |
| Intron1 | chrX:33067785-33087549 | nested | 19763 | TAgt | agTG |
| Intron1 | chrX:33064111-33067242 | nested | 3130 | GCct | acCT |
| Intron1 | chrX:33063744-33088397 | nested | 24652 | ATgt | agCT |
| Intron1 | chrX:33061488-33079879 | nested | 18390 | CTct | acCT |
| Intron1 | chrX:33059671-33059759 | nested | 87 | TTct | tgCC |
| Intron1 | chrX:33056212-33067538 | nested | 11325 | CTgt | agAA |
| Intron1 | chrX:33038317-33226522 | 3'RS | 188204 | ATgt | agAT |
| Intron1 | chrX:33038317-33142924 | 3'RS | 104606 | AGgt | agAT |
| Intron1 | chrX:33038317-33132931 | 3'RS | 94613 | TGgt | agAT |
| Intron1 | chrX:33038317-33096303 | 3'RS | 57985 | TGgt | agAT |
| Intron1 | chrX:33038317-33066589 | 3'RS | 28271 | AGgt | agAT |
| Intron 2 | chrX:33036851-33038256 | 5'RS | 1404 | AGgt | agTT |
| Intron 2 | chrX:33036719-33036776 | nested | 56 | TTct | ttTA |
| Intron 2 | chrX:33035321-33035597 | nested | 275 | TGgt | tgGC |
| Intron 2 | chrX:33028514-33031251 | nested | 2736 | ACct | acCT |
| Intron 2 | chrX:33015747-33038256 | 5'RS | 22508 | AGgt | agCT |
| Intron 2 | chrX:33004939-33032142 | nested | 27202 | AAct | acCT |
| Intron 2 | chrX:33002418-33014866 | nested | 12447 | TCac | caGC |

| | | | | | |
|---|---|---|---|---|---|
| Intron 2 | chrX:33002395-33010979 | nested | 8583 | CGtg | ctCC |
| Intron 2 | chrX:33000025-33029108 | nested | 29082 | ACct | acCT |
| Intron 2 | chrX:32994094-32996853 | nested | 2758 | CAgt | caAA |
| Intron 2 | chrX:32986900-32993632 | nested | 6731 | AAtc | agTG |
| Intron 2 | chrX:32985194-32999681 | nested | 14486 | TGct | acCT |
| Intron 2 | chrX:32984748-33006928 | nested | 22179 | AGgt | agTC |
| Intron 2 | chrX:32984486-33022658 | nested | 38171 | CTta | caCT |
| Intron 2 | chrX:32983439-33017724 | nested | 34285 | AGgc | agAT |
| Intron 2 | chrX:32983439-32986976 | nested | 3536 | AGgt | agAT |
| Intron 2 | chrX:32982877-32984525 | nested | 1647 | AGgt | agGA |
| Intron 2 | chrX:32981833-33015594 | nested | 33760 | AGgt | agGT |
| Intron 2 | chrX:32980745-33001646 | nested | 20901 | GTat | agGT |
| Intron 2 | chrX:32980613-33030315 | nested | 49701 | ACct | acCT |
| Intron 2 | chrX:32978464-33038256 | 5'RS | 59791 | AGgt | agGT |
| Intron 2 | chrX:32978004-32978325 | nested | 320 | GTgt | agTC |
| Intron 2 | chrX:32970341-32983584 | nested | 13242 | AGgc | agGA |
| Intron 2 | chrX:32970216-33000679 | nested | 30462 | TGct | acAT |
| Intron 2 | chrX:32968707-32991569 | nested | 22861 | CCct | acCT |
| Intron 2 | chrX:32966889-32966992 | nested | 102 | TTtg | agCA |
| Intron 2 | chrX:32966647-32969244 | nested | 2596 | AGct | acAA |
| Intron 2 | chrX:32961468-32993014 | nested | 31545 | CAtt | gcAT |
| Intron 2 | chrX:32960373-32990557 | nested | 30183 | TTag | atGG |
| Intron 2 | chrX:32952440-32954439 | nested | 1998 | GCct | acCT |
| Intron 2 | chrX:32949958-32965585 | nested | 15626 | TTct | acCT |
| Intron 2 | chrX:32944618-32970547 | nested | 25928 | GGct | taCT |
| Intron 2 | chrX:32943977-32973085 | nested | 29107 | ACgt | agTT |
| Intron 2 | chrX:32939983-32955800 | nested | 15816 | CCat | ggAT |
| Intron 2 | chrX:32939981-32955802 | nested | 15820 | ATcc | atGG |
| Intron 2 | chrX:32939981-32955798 | nested | 15816 | ATgc | atGG |
| Intron 2 | chrX:32936512-32941609 | nested | 5096 | TGgc | tgGG |
| Intron 2 | chrX:32928499-32929997 | nested | 1497 | ATtt | atCC |
| Intron 2 | chrX:32922709-32988728 | nested | 66018 | ACcc | tcCA |
| Intron 2 | chrX:32916732-32928991 | nested | 12258 | GGct | acCT |
| Intron 2 | chrX:32915588-32935554 | nested | 19965 | ATct | acCT |
| Intron 2 | chrX:32914109-32920266 | nested | 6156 | GTgt | caAG |
| Intron 2 | chrX:32913977-32920386 | nested | 6408 | CCac | acAC |
| Intron 2 | chrX:32913973-32920384 | nested | 6410 | ACtc | acAC |
| Intron 2 | chrX:32901485-32947426 | nested | 45940 | AGct | acCA |
| Intron 2 | chrX:32897097-33038256 | 5'RS | 141158 | AGgt | agGC |
| Intron 2 | chrX:32880929-32881730 | nested | 800 | TAgt | caGT |
| Intron 2 | chrX:32880928-32881730 | nested | 801 | TAgt | agTG |
| Intron 2 | chrX:32879459-32918246 | nested | 38786 | AAct | acAT |
| Intron 2 | chrX:32867937-32997793 | 3'RS | 129855 | AGgt | agTT |
| Intron 2 | chrX:32867937-32978325 | 3'RS | 110387 | GTgt | agTT |
| Intron 2 | chrX:32867937-32977886 | 3'RS | 109948 | TGgt | agTT |

| | | | | | |
|---|---|---|---|---|---|
| Intron 2 | chrX:32867937-32897000 | 3'RS | 29062 | AGgt | agTT |
| Intron 3 | chrX:32866362-32867845 | 5'RS | 1482 | TGgt | agAA |
| Intron 3 | chrX:32863793-32867845 | 5'RS | 4051 | TGgt | agGT |
| Intron 3 | chrX:32862977-32866258 | 3'RS | 3280 | AGgt | agCC |
| Intron 3 | chrX:32862977-32864739 | 3'RS | 1761 | AGgt | agCC |
| Intron 4 | chrX:32845333-32847873 | nested | 2539 | TGgt | gtTA |
| Intron 4 | chrX:32841504-32847873 | 3'RS | 6368 | TGgt | agGT |
| **Intron 4** | **chrX:32841504-32843308** | **3'RS** | **1803** | **GAgt** | **agGT** |
| Intron 7 | chrX:32826952-32827610 | 5'RS | 657 | AGgt | agCC |
| Intron 7 | chrX:32826743-32827610 | 5'RS | 866 | AGgt | agGA |
| Intron 7 | chrX:32823659-32827610 | 5'RS | 3950 | AGgt | agTT |
| Intron 7 | chrX:32822566-32827610 | 5'RS | 5043 | AGgt | agAG |
| Intron 7 | chrX:32821424-32825427 | nested | 4002 | TTct | acCA |
| Intron 7 | chrX:32806669-32825840 | nested | 19170 | ACct | acAG |
| Intron 7 | chrX:32789893-32804201 | nested | 14308 | CTta | atGT |
| Intron 7 | chrX:32788469-32791179 | nested | 2709 | ATag | taCT |
| Intron 7 | chrX:32770118-32771619 | nested | 1500 | GGgt | agGT |
| Intron 7 | chrX:32766076-32778941 | nested | 12864 | AGgt | agCT |
| **Intron 7** | **chrX:32752559-32827610** | **5'RS** | **75050** | **AGgt** | **agGC** |
| Intron 7 | chrX:32752559-32762979 | nested | 10419 | TGgt | agGC |
| Intron 7 | chrX:32731920-32773836 | nested | 41915 | TGct | taAG |
| Intron 7 | chrX:32717410-32826724 | 3'RS | 109313 | TGgt | agAT |
| Intron 7 | chrX:32717410-32762979 | 3'RS | 45568 | TGgt | agAT |
| Intron 7 | chrX:32717410-32752402 | 3'RS | 34991 | TGgt | agAT |
| **Intron 7** | **chrX:32717410-32752398** | **3'RS** | **34987** | **TGgt** | **agAT** |
| Intron 7 | chrX:32717410-32718228 | 3'RS | 817 | CAgt | agAT |
| Intron 9 | chrX:32696819-32713361 | nested | 16541 | TTca | ggAA |
| Intron 9 | chrX:32692100-32694159 | nested | 2058 | TGgt | agGT |
| Intron 9 | chrX:32691450-32692790 | nested | 1339 | AGgt | agTG |
| Intron 9 | chrX:32687693-32687866 | nested | 172 | AGgc | agAG |
| Intron 9 | chrX:32677952-32680545 | nested | 2592 | ATca | tcCT |
| Intron 9 | chrX:32675134-32702174 | nested | 27039 | CAct | caCA |
| Intron 9 | chrX:32664043-32715987 | 5'RS | 51943 | AGgt | agGT |
| Intron 9 | chrX:32663750-32680921 | nested | 17170 | TGct | acCT |
| Intron 9 | chrX:32663269-32690925 | 3'RS | 27655 | AGgt | agCA |
| Intron 9 | chrX:32663269-32688346 | 3'RS | 25076 | AGgt | agCA |
| Intron 9 | chrX:32663269-32664799 | 3'RS | 1529 | AAgt | agCA |
| Intron 9 | chrX:32663269-32663919 | 3'RS | 649 | GGgt | agCA |
| Intron 10 | chrX:32662973-32663081 | 5'RS | 107 | AGgt | agTT |
| Intorn 11 | chrX:32641450-32649201 | nested | 7750 | GTct | acCT |
| Intron 12 | chrX:32632160-32632420 | 5'RS | 259 | AGgt | agAA |
| Intron 12 | chrX:32632064-32632420 | 5'RS | 355 | AGgt | agGC |
| Intron 12 | chrX:32632016-32632420 | 5'RS | 403 | AGgt | agAA |
| Intron 12 | chrX:32613993-32631999 | 3'RS | 18005 | AGgt | agGT |
| Intron 12 | chrX:32613993-32621453 | 3'RS | 7459 | AGgt | agGT |

| | | | | | |
|---|---|---|---|---|---|
| Intron 13 | chrX:32600788-32613874 | 5'RS | 13085 | AGgt | agAT |
| Intron 13 | chrX:32591963-32610643 | 3'RS | 18679 | CGgt | agGT |
| Intorn 15 | chrX:32591167-32591647 | 5'RS | 479 | CCgt | agGC |
| Intron 15 | chrX:32586890-32591647 | 5'RS | 4756 | CCgt | agTT |
| Intron 15 | chrX:32585560-32586757 | nested | 1196 | AGtc | agCC |
| Intron 15 | chrX:32585552-32586621 | nested | 1068 | AGat | cgAC |
| Intron 15 | chrX:32584945-32591647 | 5'RS | 6701 | CCgt | agAT |
| Intron 15 | chrX:32583998-32591132 | 3'RS | 7133 | TGgt | agGT |
| Intron 15 | chrX:32583998-32586764 | 3'RS | 2765 | AGgt | agGT |
| Intron 15 | chrX:32583998-32584757 | 3'RS | 758 | TGgt | agGT |
| Intron 16 | chrX:32583315-32583819 | 5'RS | 503 | AGgt | agAC |
| Intron 16 | chrX:32563451-32583227 | 3'RS | 19775 | GGgt | agAT |
| Intron 17 | chrX:32562901-32563276 | 5'RS | 374 | AGgt | agTA |
| Intron 17 | chrX:32556124-32560603 | nested | 4478 | TCct | acCT |
| Intron 17 | chrX:32551926-32563276 | 5'RS | 11349 | AGgt | agGG |
| Intron 17 | chrX:32545703-32563276 | 5'RS | 17572 | AGgt | agCA |
| Intron 17 | chrX:32536248-32562797 | 3'RS | 26548 | GAgt | agGT |
| Intron 17 | chrX:32536248-32551776 | 3'RS | 15527 | TGgt | agGT |
| Intron 17 | chrX:32536248-32545567 | 3'RS | 9318 | TAgt | agGT |
| Intron 18 | chrX:32532339-32536125 | 5'RS | 3785 | ATgt | agTG |
| Intron 18 | chrX:32532339-32533049 | nested | 709 | AGgt | agTG |
| Intron 18 | chrX:32519959-32532208 | 3'RS | 12248 | AGgt | agGC |
| Intron 20 | chrX:32508669-32509394 | 5'RS | 724 | AGgt | agAA |
| Intron 20 | chrX:32503216-32508540 | 3'RS | 5323 | ATgt | agGA |
| Intron 21 | chrX:32499062-32503036 | 5'RS | 3973 | AAgt | agGC |
| Intron 21 | chrX:32490432-32503036 | 5'RS | 12603 | AAgt | tgAT |
| Intron 21 | chrX:32490426-32499222 | 3'RS | 8795 | TGgt | agTT |
| Intron 21 | chrX:32490426-32498997 | 3'RS | 8570 | AGgt | agTT |
| Intron 22 | chrX:32489392-32490281 | 5'RS | 888 | AGgt | agAC |
| Intron 22 | chrX:32486827-32489134 | 3'RS | 2306 | AGgt | agGC |
| Intron 25 | chrX:32472949-32476886 | 3'RS | 3936 | AGgt | agGT |
| Intron 26 | chrX:32471940-32472779 | 5'RS | 838 | AGgt | agGA |
| Intron 26 | chrX:32466755-32471857 | 3'RS | 5101 | GGgt | agAG |
| Intron 27 | chrX:32460395-32466573 | 5'RS | 6177 | AAgt | agAT |
| Intron 27 | chrX:32459600-32466573 | 5'RS | 6972 | AAgt | agAG |
| Intron 27 | chrX:32459431-32460277 | 3'RS | 845 | CGgt | agGA |
| Intron 28 | chrX:32457296-32457348 | nested | 51 | AGat | agAA |
| Intron 29 | chrX:32437160-32456358 | 5'RS | 19197 | AGgt | agAT |
| Intron 29 | chrX:32430488-32456358 | 5'RS | 25869 | AGgt | agGA |
| Intron 29 | chrX:32430180-32456358 | 5'RS | 26177 | AGgt | agAG |
| Intron 29 | chrX:32430180-32430279 | nested | 98 | ATgt | agAG |
| Intron 29 | chrX:32430030-32430279 | 3'RS | 248 | ATgt | agGC |
| Intron 29 | chrX:32430030-32430136 | 3'RS | 106 | AGgt | agGC |
| Intron 30 | chrX:32422551-32429869 | 5'RS | 7317 | AGgc | agAT |
| Intron 32 | chrX:32407220-32407618 | 5'RS | 397 | TGgt | agGC |

| | | | | | |
|---|---|---|---|---|---|
| Intron 32 | chrX:32404582-32407153 | 3'RS | 2570 | AGgt | agAA |
| Intron 32 | chrX:32404582-32406938 | 3'RS | 2355 | AGgt | agAA |
| **Intron 33** | **chrX:32403827-32404427** | **5'RS** | **599** | **AGgt** | **agAG** |
| **Intron 33** | **chrX:32398797-32403732** | **3'RS** | **4934** | **TAgt** | **agGT** |
| Intron 34 | chrX:32391163-32398627 | 5'RS | 7463 | AGgt | tgAT |
| **Intron 34** | **chrX:32391154-32398627** | **5'RS** | **7472** | **AGgt** | **agAG** |
| **Intron 34** | **chrX:32384877-32398627** | **5'RS** | **13749** | **AGgt** | **agGC** |
| **Intron 34** | **chrX:32383316-32391083** | **3'RS** | **7766** | **AGgt** | **agGC** |
| **Intron 34** | **chrX:32383316-32384790** | **3'RS** | **1473** | **AGgt** | **agGC** |
| Intron 37 | chrX:32379489-32379620 | nested | 130 | AGtt | agTT |
| Intron 37 | chrX:32374494-32380477 | nested | 5982 | TTgt | acTT |
| Intron 40 | chrX:32360967-32361251 | 5'RS | 283 | AGgt | agAT |
| Intron 41 | chrX:32348284-32350305 | nested | 2020 | AAgt | atAA |
| Intron 41 | chrX:32345423-32346113 | nested | 689 | GTct | acTT |
| Intron 42 | chrX:32308624-32328199 | 5'RS | 19574 | AGgt | agAA |
| **Intron 43** | **chrX:32302550-32305646** | **5'RS** | **3095** | **GGgt** | **agAG** |
| Intron 43 | chrX:32300713-32305646 | 5'RS | 4932 | GGgt | agCA |
| Intron 43 | chrX:32287674-32305646 | 5'RS | 17971 | GGgt | agTG |
| Intron 43 | chrX:32265205-32288755 | nested | 23549 | ACta | tcTT |
| Intron 43 | chrX:32262736-32276194 | nested | 13457 | CAgt | agGT |
| Intron 43 | chrX:32259793-32305646 | 5'RS | 45853 | GGgt | agAA |
| Intron 43 | chrX:32258870-32265192 | nested | 6321 | TCgt | agGA |
| Intron 43 | chrX:32253321-32305646 | 5'RS | 52324 | GGgt | agAT |
| **Intron 43** | **chrX:32243367-32301914** | **nested** | **58546** | **CAac** | **agGC** |
| Intron 43 | chrX:32241123-32258603 | nested | 17479 | TGgg | ctGG |
| Intron 43 | chrX:32241100-32258564 | nested | 17463 | TGgt | ggGT |
| Intron 43 | chrX:32235180-32287583 | 3'RS | 52402 | AGgc | agGC |
| Intron 43 | chrX:32235180-32253264 | 3'RS | 18083 | TGgt | agGC |
| Intron 44 | chrX:32231675-32233685 | nested | 2009 | AAgt | ccCC |
| Intron 44 | chrX:32225184-32227821 | nested | 2636 | TTct | acCT |
| Intron 44 | chrX:32222735-32231789 | nested | 9053 | GAcc | aaGA |
| Intron 44 | chrX:32217757-32232025 | nested | 14268 | CTct | gaCT |
| Intron 44 | chrX:32214856-32220771 | nested | 5914 | GCag | agTG |
| Intron 44 | chrX:32214840-32220770 | nested | 5929 | CAgt | gcGT |
| Intron 44 | chrX:32213994-32235033 | 5'RS | 21038 | AGgt | agCA |
| Intron 44 | chrX:32209646-32223778 | nested | 14131 | AGgt | agCT |
| Intron 44 | chrX:32206654-32206836 | nested | 181 | AAca | ggAT |
| Intron 44 | chrX:32204260-32204957 | nested | 696 | ACct | acCT |
| Intron 44 | chrX:32204071-32235033 | 5'RS | 30961 | AGgt | agAT |
| Intron 44 | chrX:32197980-32204760 | nested | 6779 | AGct | acAT |
| Intron 44 | chrX:32196292-32207143 | nested | 10850 | TCat | tcAG |
| Intron 44 | chrX:32196248-32224812 | nested | 28563 | TGgt | agAT |
| Intron 44 | chrX:32195156-32201642 | nested | 6485 | GAct | agTG |
| Intron 44 | chrX:32194159-32204068 | nested | 9908 | CTcc | ctTT |

| | | | | | |
|---|---|---|---|---|---|
| Intron 44 | chrX:32187196-32235033 | 5'RS | 47836 | AGgt | agGT |
| Intron 44 | chrX:32183938-32199023 | nested | 15084 | CCac | ccCC |
| Intron 44 | chrX:32183856-32199097 | nested | 15240 | GGat | agAT |
| Intron 44 | chrX:32183856-32199074 | nested | 15217 | GGct | agAT |
| Intron 44 | chrX:32180091-32181369 | nested | 1277 | AGgt | agGT |
| Intron 44 | chrX:32174262-32178618 | nested | 4355 | AGca | agCC |
| Intron 44 | chrX:32173725-32174801 | nested | 1075 | AGgt | agGT |
| Intron 44 | chrX:32168708-32180670 | nested | 11961 | ATct | acCT |
| Intron 44 | chrX:32152984-32180257 | nested | 27272 | AGgt | agTT |
| Intron 44 | chrX:32150342-32154477 | nested | 4134 | TTct | acCC |
| Intron 44 | chrX:32150003-32151717 | nested | 1713 | GAct | acCC |
| Intron 44 | chrX:32147136-32163923 | nested | 16786 | AGgt | agGT |
| Intron 44 | chrX:32146000-32166358 | nested | 20357 | AGgt | agCA |
| Intron 44 | chrX:32145531-32235033 | 5'RS | 89501 | AGgt | agAG |
| Intron 44 | chrX:32142992-32159795 | nested | 16802 | TTgc | acTC |
| Intron 44 | chrX:32140026-32142975 | nested | 2948 | TTcc | tgGA |
| Intron 44 | chrX:32138627-32144103 | nested | 5475 | CCta | ttCA |
| Intron 44 | chrX:32135329-32152165 | nested | 16835 | AGgt | agCT |
| Intron 44 | chrX:32130935-32141736 | nested | 10800 | AGgt | agGA |
| Intron 44 | chrX:32130749-32130870 | nested | 120 | TAgc | agGT |
| Intron 44 | chrX:32130683-32130736 | nested | 52 | CCct | tcCT |
| Intron 44 | chrX:32130678-32142421 | nested | 11742 | AGct | acCA |
| Intron 44 | chrX:32125559-32159797 | nested | 34237 | TGtt | gtTG |
| Intron 44 | chrX:32119631-32119735 | nested | 103 | GTaa | ggAA |
| Intron 44 | chrX:32114108-32130677 | nested | 16568 | CAtt | acCA |
| Intron 44 | chrX:32106615-32235033 | 5'RS | 128417 | AGgt | agTC |
| Intron 44 | chrX:32104925-32107813 | nested | 2887 | GCct | acCT |
| Intron 44 | chrX:32104838-32105024 | nested | 185 | AGaa | agAC |
| Intron 44 | chrX:32103390-32125453 | nested | 22062 | ATaa | atGT |
| Intron 44 | chrX:32101175-32136722 | nested | 35546 | AGgt | agGG |
| Intron 44 | chrX:32100282-32149259 | nested | 48976 | GCac | ggAA |
| Intron 44 | chrX:32099434-32235033 | 5'RS | 135598 | AGgt | agTA |
| Intron 44 | chrX:32086171-32110213 | nested | 24041 | TCct | acCC |
| Intron 44 | chrX:32057841-32086765 | nested | 28923 | AGgt | agGA |
| Intron 44 | chrX:32013363-32041279 | nested | 27915 | AAga | ttTT |
| Intron 44 | chrX:32012574-32013670 | nested | 1095 | TTct | tgGT |
| Intron 44 | chrX:32012423-32039018 | nested | 26594 | GGgt | agGC |
| Intron 44 | chrX:32002687-32026251 | nested | 23563 | TAct | acTT |
| Intron 44 | chrX:31997618-32024204 | nested | 26585 | GTct | acCA |
| Intron 44 | chrX:31986631-32145384 | 3'RS | 158752 | AGgt | agGA |
| Intron 44 | chrX:31986631-32032701 | 3'RS | 46069 | AGgt | agGA |
| Intron 44 | chrX:31986631-32011526 | 3'RS | 24894 | AGgt | agGA |
| Intron 44 | chrX:31986631-31989924 | 3'RS | 3292 | TGgt | agGA |
| Intron 45 | chrX:31982744-31985412 | nested | 2667 | CCtt | atCA |
| Intron 45 | chrX:31980251-31986456 | 5'RS | 6204 | AGgt | agGA |

| | | | | | |
|---|---|---|---|---|---|
| Intron 45 | chrX:31957456-31986456 | 5'RS | 28999 | AGgt | agGA |
| Intron 45 | chrX:31950344-31980093 | 3'RS | 29748 | TGgt | agGC |
| Intron 45 | chrX:31950344-31968950 | 3'RS | 18605 | AGgt | agGC |
| Intron 45 | chrX:31950344-31957379 | 3'RS | 7034 | AGgt | agGC |
| Intron 47 | chrX:31946313-31947713 | 5'RS | 1399 | AGgt | agAT |
| Intron 47 | chrX:31928802-31947713 | 5'RS | 18910 | AGgt | agAG |
| Intron 47 | chrX:31897296-31924445 | nested | 27148 | Caca | ctCT |
| Intron 47 | chrX:31893490-31895495 | 3'RS | 2004 | AGgt | agGT |
| Intron 49 | chrX:31851409-31851477 | nested | 67 | CTtt | ccCT |
| Intron 50 | chrX:31821027-31838092 | 5'RS | 17064 | CTgt | agGG |
| Intron 50 | chrX:31792309-31820915 | 3'RS | 28605 | AGgc | agCT |
| Intron 50 | chrX:31792309-31802421 | 3'RS | 10111 | AGgt | agCT |
| Intron 51 | chrX:31770641-31792077 | 5'RS | 21435 | AGgt | agGG |
| Intron 51 | chrX:31766714-31766806 | nested | 91 | ATct | taAT |
| Intron 51 | chrX:31757843-31773392 | nested | 15548 | AGct | acCT |
| Intron 51 | chrX:31747865-31770558 | 3'RS | 22692 | TGgt | agGC |
| Intron 52 | chrX:31717055-31744267 | nested | 27211 | AGgt | agTC |
| Intron 53 | chrX:31694386-31697492 | 5'RS | 3105 | AGgt | agGA |
| Intron 53 | chrX:31691721-31697492 | 5'RS | 5770 | AGgt | agAT |
| **Intron 53** | **chrX:31687955-31697492** | **5'RS** | **9536** | **AGgt** | **agAA** |
| Intron 53 | chrX:31676261-31694324 | 3'RS | 18062 | CCgt | agCA |
| Intron 53 | chrX:31676261-31691642 | 3'RS | 15380 | AAgt | agCA |
| Intron 53 | chrX:31676261-31679560 | 3'RS | 3298 | AGgt | agCA |
| Intron 55 | chrX:31635438-31638786 | nested | 3347 | GGct | acCT |
| Intron 55 | chrX:31635408-31638799 | nested | 3390 | CAgc | caAA |
| Intron 55 | chrX:31635332-31641654 | nested | 6321 | TGta | atTA |
| Intron 55 | chrX:31602203-31602783 | nested | 579 | TGtt | agTT |
| Intron 55 | chrX:31602195-31602815 | nested | 619 | AAct | tcCT |
| Intron 55 | chrX:31602194-31602790 | nested | 595 | Ccat | ccTT |
| Intron 55 | chrX:31600126-31601887 | nested | 1760 | TAat | taAT |
| Intron 55 | chrX:31600122-31601883 | nested | 1760 | AAaa | caCA |
| Intron 55 | chrX:31595644-31633324 | nested | 37679 | AGgt | agGC |
| Intron 55 | chrX:31569054-31570557 | nested | 1502 | CAgt | caAA |
| Intron 56 | chrX:31515061-31515196 | 3'RS | 134 | AAgt | agGT |
| Intron 59 | chrX:31494156-31496223 | 5'RS | 2066 | AGgt | agAG |
| Intron 59 | chrX:31481349-31484903 | nested | 3553 | GAta | ttAA |
| Intron 59 | chrX:31462744-31494069 | 3'RS | 31325 | AGgt | agGC |
| Intron 60 | chrX:31436609-31456046 | nested | 19436 | TAgt | agTA |
| Intron 60 | chrX:31432541-31442323 | nested | 9781 | CAgt | agCT |
| Intron 60 | chrX:31415859-31433414 | nested | 17554 | AAgt | agCC |
| Intron 60 | chrX:31396436-31422093 | nested | 25656 | AAct | acCT |
| Intron 60 | chrX:31384586-31399584 | nested | 14997 | AGgt | tgAG |
| Intron 62 | chrX:31303503-31341715 | 5'RS | 38211 | AAgt | agTT |
| Intron 62 | chrX:31295455-31314637 | nested | 19181 | CAgt | agGC |
| Intron 62 | chrX:31288091-31341715 | 5'RS | 53623 | AAgt | agGC |

| | | | | | |
|---|---|---|---|---|---|
| Intron 62 | chrX:31279133-31284927 | 3'RS | 5793 | GGgt | agCC |
| Intron 62 | chrX:31279133-31283560 | 3'RS | 4426 | TGgt | agCC |
| Intron 63 | chrX:31270868-31274191 | nested | 3322 | GGct | gcGT |
| Intron 63 | chrX:31265885-31279072 | 5'RS | 13186 | AGgt | agTT |
| Intron 63 | chrX:31243757-31243912 | nested | 154 | GAgt | agGT |
| Intron 63 | chrX:31241238-31265811 | 3'RS | 24572 | TGgt | agCT |
| Intron 67 | chrX:31221511-31222078 | 5'RS | 566 | TTgt | agTC |
| Intron 67 | chrX:31217814-31222078 | 5'RS | 4263 | TTgt | agGT |
| Intron 67 | chrX:31217550-31217667 | nested | 116 | AGga | agGA |
| Intron 67 | chrX:31213762-31222078 | 5'RS | 8315 | TTgt | agTC |
| Intron 67 | chrX:31201021-31216719 | 3'RS | 15697 | AGgt | agGC |
| Intron 74 | chrX:31173469-31183019 | nested | 9559 | ACct | acCT |
| Intron 74 | chrX:31165635-31166438 | 3'RS | 802 | TGgt | agGA |
| Intron 77 | chrX:31150750-31152219 | 5'RS | 1468 | AGgt | agAT |
| Intron 77 | chrX:31144916-31152219 | 5'RS | 7302 | AGgt | agCA |
| Intron 77 | chrX:31144790-31150600 | 3'RS | 5809 | AGgt | agGA |

**Table S5. Overview of single and multi-step splicing of *DMD* introns.**

| DP427m | Start | Stop | Intron length | Splicing |
|---|---|---|---|---|
| Intron 1 | 33,229,398 | 33,038,318 | 191,081 | Multi-step |
| Intron 2 | 33,038,255 | 32,867,938 | 170,318 | Multi-step |
| Intron 3 | 32,867,844 | 32,862,978 | 4,867 | Multi-step |
| Intron 4 | 32,862,899 | 32,841,505 | 21,395 | Multi-step |
| Intron 5 | 32,841,411 | 32,834,758 | 6,654 | Single step |
| Intron 6 | 32,834,584 | 32,827,729 | 6,856 | Single step |
| Intron 7 | 32,827,609 | 32,717,411 | 110,199 | Multi-step |
| Intron 8 | 32,717,228 | 32,716,116 | 1,113 | Single step |
| Intron 9 | 32,715,986 | 32,663,270 | 52,717 | Multi-step |
| Intron 10 | 32,663,080 | 32,662,431 | 650 | Multi-step |
| Intron 11 | 32,662,248 | 32,632,571 | 29,678 | Multi-step |
| Intron 12 | 32,632,419 | 32,613,994 | 18,426 | Multi-step |
| Intron 13 | 32,613,873 | 32,591,964 | 21,910 | Multi-step |
| Intron 14 | 32,591,861 | 32,591,755 | 107 | Single step |
| Intron 15 | 32,591,646 | 32,583,999 | 7,648 | Multi-step |
| Intron 16 | 32,583,818 | 32,563,452 | 20,367 | Multi-step |
| Intron 17 | 32,563,275 | 32,536,249 | 27,027 | Multi-step |
| Intron 18 | 32,536,124 | 32,519,960 | 16,165 | Multi-step |
| Intron 19 | 32,519,871 | 32,509,636 | 10,236 | Single step |
| Intron 20 | 32,509,393 | 32,503,217 | 6,177 | Multi-step |
| Intron 21 | 32,503,035 | 32,490,427 | 12,609 | Multi-step |
| Intron 22 | 32,490,280 | 32,486,828 | 3,453 | Multi-step |
| Intron 23 | 32,486,614 | 32,482,817 | 3,798 | Single step |
| Intron 24 | 32,482,702 | 32,481,712 | 991 | Single step |
| Intron 25 | 32,481,555 | 32,472,950 | 8,606 | Multi-step |
| Intron 26 | 32,472,778 | 32,466,756 | 6,023 | Multi-step |
| Intron 27 | 32,466,572 | 32,459,432 | 7,141 | Multi-step |
| Intron 28 | 32,459,296 | 32,456,508 | 2,789 | Multi-step |
| Intron 29 | 32,456,357 | 32,430,031 | 26,327 | Multi-step |
| Intron 30 | 32,429,868 | 32,408,299 | 21,570 | Multi-step |
| Intron 31 | 32,408,187 | 32,407,792 | 396 | Single step |
| Intron 32 | 32,407,617 | 32,404,583 | 3,035 | Multi-step |
| Intron 33 | 32,404,426 | 32,398,798 | 5,629 | Multi-step |
| Intron 34 | 32,398,626 | 32,383,317 | 15,310 | Multi-step |
| Intron 35 | 32,383,136 | 32,382,828 | 309 | Single step |
| Intron 36 | 32,382,698 | 32,381,076 | 1,623 | Single step |
| Intron 37 | 32,380,904 | 32,366,646 | 14,259 | Multi-step |
| Intron 38 | 32,366,522 | 32,364,198 | 2,325 | Single step |
| Intron 39 | 32,364,059 | 32,361,404 | 2,656 | Single step |
| Intron 40 | 32,361,250 | 32,360,400 | 851 | Single step |
| Intron 41 | 32,360,216 | 32,328,394 | 31,823 | Multi-step |
| Intron 42 | 32,328,198 | 32,305,819 | 22,380 | Multi-step |
| Intron 43 | 32,305,645 | 32,235,181 | 70,465 | Multi-step |
| Intron 44 | 32,235,032 | 31,986,632 | 248,401 | Multi-step |
| Intron 45 | 31,986,455 | 31,950,345 | 36,111 | Multi-step |
| Intron 46 | 31,950,196 | 31,947,863 | 2,334 | Single step |

| | | | | |
|---|---|---|---|---|
| Intron 47 | 31,947,712 | 31,893,491 | 54,222 | Multi-step |
| Intron 48 | 31,893,304 | 31,854,937 | 38,368 | Single step |
| Intron 49 | 31,854,834 | 31,838,201 | 16,634 | Multi-step |
| Intron 50 | 31,838,091 | 31,792,310 | 45,782 | Multi-step |
| Intron 51 | 31,792,076 | 31,747,866 | 44,211 | Multi-step |
| Intron 52 | 31,747,747 | 31,697,704 | 50,044 | Multi-step |
| Intron 53 | 31,697,491 | 31,676,262 | 21,230 | Multi-step |
| Intron 54 | 31,676,106 | 31,645,980 | 30,127 | Single step |
| Intron 55 | 31,645,789 | 31,525,571 | 120,219 | Multi-step |
| Intron 56 | 31,525,397 | 31,515,062 | 10,336 | Multi-step |
| Intron 57 | 31,514,904 | 31,497,221 | 17,684 | Single step |
| Intron 58 | 31,497,099 | 31,496,492 | 608 | Single step |
| Intron 59 | 31,496,222 | 31,462,745 | 33,478 | Multi-step |
| Intron 60 | 31,462,597 | 31,366,752 | 95,846 | Multi-step |
| Intron 61 | 31,366,672 | 31,341,776 | 24,897 | Single step |
| Intron 62 | 31,341,714 | 31,279,134 | 62,581 | Multi-step |
| Intron 63 | 31,279,071 | 31,241,239 | 37,833 | Multi-step |
| Intron 64 | 31,241,163 | 31,227,817 | 13,347 | Single step |
| Intron 65 | 31,227,614 | 31,224,785 | 2,830 | Single step |
| Intron 66 | 31,224,698 | 31,222,236 | 2,463 | Single step |
| Intron 67 | 31,222,077 | 31,201,022 | 21,056 | Multi-step |
| Intron 68 | 31,200,854 | 31,198,599 | 2,256 | Single step |
| Intron 69 | 31,198,486 | 31,196,923 | 1,564 | Single step |
| Intron 70 | 31,196,785 | 31,196,088 | 698 | Single step |
| Intron 71 | 31,196,048 | 31,191,722 | 4,327 | Single step |
| Intron 72 | 31,191,655 | 31,190,531 | 1,125 | Single step |
| Intron 73 | 31,190,464 | 31,187,719 | 2,746 | Single step |
| Intron 74 | 31,187,559 | 31,165,636 | 21,924 | Multi-step |
| Intron 75 | 31,165,391 | 31,164,532 | 860 | Single step |
| Intron 76 | 31,164,407 | 31,152,312 | 12,096 | Single step |
| Intron 77 | 31,152,218 | 31,144,791 | 7,428 | Multi-step |
| Intron 78 | 31,144,758 | 31,140,048 | 4,711 | Single step |

**Table S6. Genomic positions (genomic ref. seq ID NG 012232.1 ) of *DMD* exons and introns, related exon and intron length used in the analysis.**

| | | Exon | | | | Intron | | |
|---|---|---|---|---|---|---|---|---|
| **DP427m** | **start** | **stop** | **length** | **DP427m** | **start** | **stop** | **length** | **length - RM** |
| Exon 1 | 33,229,636 | 33,229,399 | 238 | Intron 1 | 33,229,398 | 33,038,318 | 191,081 | 117,470 |
| Exon 2 | 33,038,317 | 33,038,256 | 62 | Intron 2 | 33,038,255 | 32,867,938 | 170,318 | 97,819 |
| Exon 3 | 32,867,937 | 32,867,845 | 93 | Intron 3 | 32,867,844 | 32,862,978 | 4,867 | 3,929 |
| Exon 4 | 32,862,977 | 32,862,900 | 78 | Intron 4 | 32,862,899 | 32,841,505 | 21,395 | 11,876 |
| Exon 5 | 32,841,504 | 32,841,412 | 93 | Intron 5 | 32,841,411 | 32,834,758 | 6,654 | 4,996 |
| Exon 6 | 32,834,757 | 32,834,585 | 173 | Intron 6 | 32,834,584 | 32,827,729 | 6,856 | 3,813 |
| Exon 7 | 32,827,728 | 32,827,610 | 119 | Intron 7 | 32,827,609 | 32,717,411 | 110,199 | 54,075 |
| Exon 8 | 32,717,410 | 32,717,229 | 182 | Intron 8 | 32,717,228 | 32,716,116 | 1,113 | 1,010 |
| Exon 9 | 32,716,115 | 32,715,987 | 129 | Intron 9 | 32,715,986 | 32,663,270 | 52,717 | 32,391 |
| Exon 10 | 32,663,269 | 32,663,081 | 189 | Intron 10 | 32,663,080 | 32,662,431 | 650 | 384 |
| Exon 11 | 32,662,430 | 32,662,249 | 182 | Intron 11 | 32,662,248 | 32,632,571 | 29,678 | 12,660 |
| Exon 12 | 32,632,570 | 32,632,420 | 151 | Intron 12 | 32,632,419 | 32,613,994 | 18,426 | 11,219 |
| Exon 13 | 32,613,993 | 32,613,874 | 120 | Intron 13 | 32,613,873 | 32,591,964 | 21,910 | 10,496 |
| Exon 14 | 32,591,963 | 32,591,862 | 102 | Intron 14 | 32,591,861 | 32,591,755 | 107 | 107 |
| Exon 15 | 32,591,754 | 32,591,647 | 108 | Intron 15 | 32,591,646 | 32,583,999 | 7,648 | 5,234 |
| Exon 16 | 32,583,998 | 32,583,819 | 180 | Intron 16 | 32,583,818 | 32,563,452 | 20,367 | 9,674 |
| Exon 17 | 32,563,451 | 32,563,276 | 176 | Intron 17 | 32,563,275 | 32,536,249 | 27,027 | 14,533 |
| Exon 18 | 32,536,248 | 32,536,125 | 124 | Intron 18 | 32,536,124 | 32,519,960 | 16,165 | 7,040 |
| Exon 19 | 32,519,959 | 32,519,872 | 88 | Intron 19 | 32,519,871 | 32,509,636 | 10,236 | 9,583 |
| Exon 20 | 32,509,635 | 32,509,394 | 242 | Intron 20 | 32,509,393 | 32,503,217 | 6,177 | 3,157 |
| Exon 21 | 32,503,216 | 32,503,036 | 181 | Intron 21 | 32,503,035 | 32,490,427 | 12,609 | 7,325 |
| Exon 22 | 32,490,426 | 32,490,281 | 146 | Intron 22 | 32,490,280 | 32,486,828 | 3,453 | 2,624 |
| Exon 23 | 32,486,827 | 32,486,615 | 213 | Intron 23 | 32,486,614 | 32,482,817 | 3,798 | 2,446 |
| Exon 24 | 32,482,816 | 32,482,703 | 114 | Intron 24 | 32,482,702 | 32,481,712 | 991 | 756 |
| Exon 25 | 32,481,711 | 32,481,556 | 156 | Intron 25 | 32,481,555 | 32,472,950 | 8,606 | 3,793 |
| Exon 26 | 32,472,949 | 32,472,779 | 171 | Intron 26 | 32,472,778 | 32,466,756 | 6,023 | 3,035 |
| Exon 27 | 32,466,755 | 32,466,573 | 183 | Intron 27 | 32,466,572 | 32,459,432 | 7,141 | 4,943 |
| Exon 28 | 32,459,431 | 32,459,297 | 135 | Intron 28 | 32,459,296 | 32,456,508 | 2,789 | 2,332 |
| Exon 29 | 32,456,507 | 32,456,358 | 150 | Intron 29 | 32,456,357 | 32,430,031 | 26,327 | 16,655 |
| Exon 30 | 32,430,030 | 32,429,869 | 162 | Intron 30 | 32,429,868 | 32,408,299 | 21,570 | 13,246 |
| Exon 31 | 32,408,298 | 32,408,188 | 111 | Intron 31 | 32,408,187 | 32,407,792 | 396 | 396 |
| Exon 32 | 32,407,791 | 32,407,618 | 174 | Intron 32 | 32,407,617 | 32,404,583 | 3,035 | 2,650 |
| Exon 33 | 32,404,582 | 32,404,427 | 156 | Intron 33 | 32,404,426 | 32,398,798 | 5,629 | 4,445 |
| Exon 34 | 32,398,797 | 32,398,627 | 171 | Intron 34 | 32,398,626 | 32,383,317 | 15,310 | 11,828 |
| Exon 35 | 32,383,316 | 32,383,137 | 180 | Intron 35 | 32,383,136 | 32,382,828 | 309 | 309 |
| Exon 36 | 32,382,827 | 32,382,699 | 129 | Intron 36 | 32,382,698 | 32,381,076 | 1,623 | 1,623 |
| Exon 37 | 32,381,075 | 32,380,905 | 171 | Intron 37 | 32,380,904 | 32,366,646 | 14,259 | 11,776 |
| Exon 38 | 32,366,645 | 32,366,523 | 123 | Intron 38 | 32,366,522 | 32,364,198 | 2,325 | 2,325 |
| Exon 39 | 32,364,197 | 32,364,060 | 138 | Intron 39 | 32,364,059 | 32,361,404 | 2,656 | 2,656 |
| Exon 40 | 32,361,403 | 32,361,251 | 153 | Intron 40 | 32,361,250 | 32,360,400 | 851 | 630 |
| Exon 41 | 32,360,399 | 32,360,217 | 183 | Intron 41 | 32,360,216 | 32,328,394 | 31,823 | 21,574 |
| Exon 42 | 32,328,393 | 32,328,199 | 195 | Intron 42 | 32,328,198 | 32,305,819 | 22,380 | 14,827 |
| Exon 43 | 32,305,818 | 32,305,646 | 173 | Intron 43 | 32,305,645 | 32,235,181 | 70,465 | 34,667 |

| | | | | | | | | |
|---|---|---|---|---|---|---|---|---|
| Exon 44 | 32,235,180 | 32,235,033 | 148 | Intron 44 | 32,235,032 | 31,986,632 | 248,401 | 162,105 |
| Exon 45 | 31,986,631 | 31,986,456 | 176 | Intron 45 | 31,986,455 | 31,950,345 | 36,111 | 16,723 |
| Exon 46 | 31,950,344 | 31,950,197 | 148 | Intron 46 | 31,950,196 | 31,947,863 | 2,334 | 1,661 |
| Exon 47 | 31,947,862 | 31,947,713 | 150 | Intron 47 | 31,947,712 | 31,893,491 | 54,222 | 30,269 |
| Exon 48 | 31,893,490 | 31,893,305 | 186 | Intron 48 | 31,893,304 | 31,854,937 | 38,368 | 20,216 |
| Exon 49 | 31,854,936 | 31,854,835 | 102 | Intron 49 | 31,854,834 | 31,838,201 | 16,634 | 8,314 |
| Exon 50 | 31,838,200 | 31,838,092 | 109 | Intron 50 | 31,838,091 | 31,792,310 | 45,782 | 24,729 |
| Exon 51 | 31,792,309 | 31,792,077 | 233 | Intron 51 | 31,792,076 | 31,747,866 | 44,211 | 26,936 |
| Exon 52 | 31,747,865 | 31,747,748 | 118 | Intron 52 | 31,747,747 | 31,697,704 | 50,044 | 25,708 |
| Exon 53 | 31,697,703 | 31,697,492 | 212 | Intron 53 | 31,697,491 | 31,676,262 | 21,230 | 10,111 |
| Exon 54 | 31,676,261 | 31,676,107 | 155 | Intron 54 | 31,676,106 | 31,645,980 | 30,127 | 21,346 |
| Exon 55 | 31,645,979 | 31,645,790 | 190 | Intron 55 | 31,645,789 | 31,525,571 | 120,219 | 72,970 |
| Exon 56 | 31,525,570 | 31,525,398 | 173 | Intron 56 | 31,525,397 | 31,515,062 | 10,336 | 7,634 |
| Exon 57 | 31,515,061 | 31,514,905 | 157 | Intron 57 | 31,514,904 | 31,497,221 | 17,684 | 10,181 |
| Exon 58 | 31,497,220 | 31,497,100 | 121 | Intron 58 | 31,497,099 | 31,496,492 | 608 | 608 |
| Exon 59 | 31,496,491 | 31,496,223 | 269 | Intron 59 | 31,496,222 | 31,462,745 | 33,478 | 18,995 |
| Exon 60 | 31,462,744 | 31,462,598 | 147 | Intron 60 | 31,462,597 | 31,366,752 | 95,846 | 51,251 |
| Exon 61 | 31,366,751 | 31,366,673 | 79 | Intron 61 | 31,366,672 | 31,341,776 | 24,897 | 15,071 |
| Exon 62 | 31,341,775 | 31,341,715 | 61 | Intron 62 | 31,341,714 | 31,279,134 | 62,581 | 41,144 |
| Exon 63 | 31,279,133 | 31,279,072 | 62 | Intron 63 | 31,279,071 | 31,241,239 | 37,833 | 24,112 |
| Exon 64 | 31,241,238 | 31,241,164 | 75 | Intron 64 | 31,241,163 | 31,227,817 | 13,347 | 9,071 |
| Exon 65 | 31,227,816 | 31,227,615 | 202 | Intron 65 | 31,227,614 | 31,224,785 | 2,830 | 2,289 |
| Exon 66 | 31,224,784 | 31,224,699 | 86 | Intron 66 | 31,224,698 | 31,222,236 | 2,463 | 1,954 |
| Exon 67 | 31,222,235 | 31,222,078 | 158 | Intron 67 | 31,222,077 | 31,201,022 | 21,056 | 11,484 |
| Exon 68 | 31,201,021 | 31,200,855 | 167 | Intron 68 | 31,200,854 | 31,198,599 | 2,256 | 2,115 |
| Exon 69 | 31,198,598 | 31,198,487 | 112 | Intron 69 | 31,198,486 | 31,196,923 | 1,564 | 906 |
| Exon 70 | 31,196,922 | 31,196,786 | 137 | Intron 70 | 31,196,785 | 31,196,088 | 698 | 227 |
| Exon 71 | 31,196,087 | 31,196,049 | 39 | Intron 71 | 31,196,048 | 31,191,722 | 4,327 | 4,327 |
| Exon 72 | 31,191,721 | 31,191,656 | 66 | Intron 72 | 31,191,655 | 31,190,531 | 1,125 | 1,125 |
| Exon 73 | 31,190,530 | 31,190,465 | 66 | Intron 73 | 31,190,464 | 31,187,719 | 2,746 | 2,746 |
| Exon 74 | 31,187,718 | 31,187,560 | 159 | Intron 74 | 31,187,559 | 31,165,636 | 21,924 | 15,064 |
| Exon 75 | 31,165,635 | 31,165,392 | 244 | Intron 75 | 31,165,391 | 31,164,532 | 860 | 860 |
| Exon 76 | 31,164,531 | 31,164,408 | 124 | Intron 76 | 31,164,407 | 31,152,312 | 12,096 | 7,643 |
| Exon 77 | 31,152,311 | 31,152,219 | 93 | Intron 77 | 31,152,218 | 31,144,791 | 7,428 | 6,681 |
| Exon 78 | 31,144,790 | 31,144,759 | 32 | Intron 78 | 31,144,758 | 31,140,048 | 4,711 | 4,467 |
| Exon 79 | 31,140,047 | 31,137,345 | 2,703 | | | | | |
